# Seroprevalence Estimates of Latent and Acute *Toxoplasma* Infections in HIV^+^ People—Call for Action in Underprivileged Communities

**DOI:** 10.3390/microorganisms9102034

**Published:** 2021-09-26

**Authors:** Ali Rostami, Seyed Mohammad Riahi, Shayan Abdollahzadeh Sagha, Ali Taghipour, Mahdi Sepidarkish, Mousa Mohammadnia-Afrouzi, Soheil Ebrahimpour, Peter J. Hotez, Ray Gamble, Robin B. Gasser

**Affiliations:** 1 Infectious Diseases and Tropical Medicine Research Center, Health Research Institute, Babol University of Medical Sciences, Babol, Iran; shayan.abdolahzadesagha@gmail.com (S.A.S.); drsoheil.d@gmail.com (S.E.); 2 Immunoregulation Research Center, Health Research Institute, Babol University of Medical Sciences, Babol, Iran; mousamohammadnia@yahoo.com; 3 Cardiovascular Diseases Research Center, Department of Epidemiology and Biostatistics, School of Medicine, Birjand University of Medical Sciences, Birjand, Iran; riahim61@gmail.com; 4 Zoonoses Research Center, School of Medicine, Jahrom University of Medical Sciences, Jahrom, Iran; alitaghipor71@yahoo.com; 5 Department of Biostatistics and Epidemiology, School of Public Health, Babol University of Medical Sciences, Babol, Iran; mahdi.sepidarkish@gmail.com; 6 Texas Children’s Center for Vaccine Development, Department of Pediatrics and Molecular Virology and Microbiology, National School of Tropical Medicine, Baylor College of Medicine, Houston, TX 77030, USA; hotez@bcm.edu; 7 National Academy of Sciences, Washington, DC 20001, USA; RGamble@nas.edu; 8 Department of Veterinary Biosciences, Melbourne Veterinary School, The University of Melbourne, Parkville, VIC 3010, Australia

**Keywords:** HIV-infected people, latent and acute *Toxoplasma gondii* infections, toxoplasmosis, global seroprevalence, systematic review, meta-analysis

## Abstract

We undertook a comprehensive, systematic review of observational studies to estimate respective seroprevalences of latent and acute *Toxoplasma gondii* infections in HIV^+^ people at the global, regional and country levels; related seroprevalence to socio-economic variables and CD4+ cell counts; and assessed temporal changes in prevalence and risk factors for this group. We systematically searched international databases for seroepidemiological surveys between 1 January 1980 and 31 July 2020. We used a random effects model to calculate pooled seroprevalences with 95% confidence intervals (CI), and estimated the numbers of HIV^+^ people inferred to harbour latent and acute *T. gondii* infections (LT or AT). We grouped seroprevalence data according to the geographic regions defined by the World Health Organization (WHO) and conducted subgroup and meta-regression analyses of the data. Of a total of 4024 studies identified, 150 and 65 of them met the inclusion criteria for LT and AT in HIV^+^ people, respectively. The overall, pooled seroprevalences of LT and AT were 37.4% (95% CI, 33.4–41.4) and 1.3% (95% CI, 0.9–1.8%), equating to ~14.2 and 0.5 million HIV^+^ people, respectively. Most HIV^+^ people with *T. gondii* infections originated from Africa, and the highest seroprevalences were in low-income countries with low human development indices. Significant risk factors for toxoplasmosis in HIV^+^ patients included the consumption of raw/undercooked meat, frequent contact with soil, a low CD4+ T lymphocyte number (<200 cells per μL) and age. Overall, the finding of high seroprevalences of particularly latent *T. gondii* infection in HIV^+^ people in underprivileged regions of the world, such as parts of Africa, calls for preventative action. Programs that include routine serological monitoring, counselling, care, animal control and/or prophylactic treatment measures are needed to prevent severe toxoplasmosis from developing in people living with HIV infection. Our study highlights the potential importance of parasite chemoprophylaxis in resource-poor settings, particularly in low-income countries.

## 1. Introduction

*Toxoplasma gondii* infection can lead to life-threating toxoplasmosis in human immunodeficiency virus (HIV)-infected people [1]. In HIV^+^ people, ‘toxoplasmic encephalitis’ is a common complication, particularly in individuals with CD4+ T lymphocyte counts of <200 cells per μL [2,3]. This form of toxoplasmosis can be associated with headache, drowsiness, disorientation, hemiparesis, retinochoroiditis and/or epilepsy [4,5], and can become generalised and fatal.

Primary *T. gondii* infection is acquired through the ingestion of sporulated oocysts in soil or water contaminated with cat faeces, or on contaminated fruits and vegetables; the ingestion of infective stages (‘zoites) within raw or undercooked meats; by vertical transmission from an infected mother to the foetus; and, in rare cases, by blood transfusion or organ transplantation [6,7,8,9,10]. While primary *T. gondii* infection is mostly asymptomatic in immunocompetent people [11], such an infection could lead to severe toxoplasmosis in HIV^+^ people due to their immunodeficiency [12]. More often, however, toxoplasmosis appears to result from the reactivation of a latent *T. gondii* infection [5,13,14]. Although a low CD4+ T cell count (<200 per µL) is a risk factor for opportunistic infections in HIV^+^ people, severe toxoplasmosis occurs in people with advanced AIDS [13], when CD8 T cell deficiency allows a reactivation of latent *T. gondii* infection [14].

Serological tools are useful to monitor and characterise *T. gondii* infections. Specific anti-*Toxoplasma* IgM and/or IgG serum antibody levels (titres) provide an indication of the status of a *T. gondii* infection and its clinical relevance. Latent infection (sometimes referred to as ‘latent toxoplasmosis’—although not always a disease state) [9] is usually associated with the presence of relatively low levels of specific IgG serum antibodies, whereas a subacute or acute infection linked to disease (‘acute toxoplasmosis’) [9,15] is associated with an initial increase in specific anti-*Toxoplasma* IgM serum antibody, followed by a four to 16-fold increase in specific IgG serum antibody over a period of 2–4 weeks [7,16,17]. Thus, serological tools underpin epidemiological investigations of the prevalence, status and/or dynamics of *T. gondii* infection and toxoplasmosis in human populations.

Estimating the prevalence and distribution of *T. gondii* infection using serodiagnostic tools and assessing associated risk factors for HIV^+^ people will assist the development and implementation of measures/programs to prevent toxoplasmosis in this risk group [18]. Previous investigations to estimate the prevalence of *T. gondii* infections in HIV^+^ people were limited to particular countries or geographical regions over short study periods (usually one year) [19,20,21,22,23]. Perhaps the most comprehensive investigation of HIV^+^ people was a meta-analysis of studies published from 1987 to 2016 [24], which considered latent infection/toxoplasmosis, but not acute toxoplasmosis; furthermore, it did not assess risk factors linked to toxoplasmosis and its prevalence.

In the present study, we undertook a comprehensive, systematic review of observational studies to (i) estimate respective seroprevalence rates for latent and acute *T. gondii* infections in HIV^+^ people at the global, regional and country levels; (ii) relate prevalence to socio-economic variables and CD4+ cell counts; and (iii) assess temporal changes in prevalence as well as risk factors for this group of people.

## 2. Materials and Methods

We followed the recommendations established by the Preferred Reporting Items for Systematic Reviews and Meta-Analyses (PRISMA) [25]. All procedures, including the literature search, the critical appraisals of full articles/reports and of data quality as well as the analyses of data were performed by experienced researchers. Disagreements regarding study selection, data extraction and quality assessment were resolved in discussion with the first author (A.R.) to reach a consensus decision.

### 2.1. Search Strategy and Selection Criteria

A systematic review of the literature was performed using PubMed (through OVID), Scopus, Web of Science, SciELO and Embase to identify all peer-reviewed articles reporting data on the prevalence of latent and acute *T. gondii* infections in HIV^+^ people, published between January 1980 and July 2020, without language or geographical restriction. We used relevant keywords for the searching of databases (Figure S1). The search strategy was discussed with an expert medical librarian for optimal inclusion-sensitivity. To ensure that we identified ‘grey’ literature, such as papers in languages other than English, conference proceedings, and technical or governmental reports, we searched the Google Scholar database, and scrutinised reference lists of reviews and original articles.

The inclusion criteria were: (i) observational studies with original data used to estimate the seroprevalence of latent or acute *T. gondii* infection/toxoplasmosis in HIV^+^ people; (ii) studies with a sample size of >30 individuals; and (iii) investigations to measure specific anti-*Toxoplasma* serum antibodies (IgM and/or IgG) or *Toxoplasma* antigens. For case-control studies, data were extracted only for HIV^+^ individuals. Excluded were studies that (a) were not representative of a ‘general population’ of people with HIV infection (e.g., encephalitis cases, particularly those with toxoplasmic encephalitis, psychiatric patients and drug users); (b) lacked a detailed description of serodiagnostic method(s) used; (c) were comparative investigations of diagnostic methods; (d) had overlapping participation in multiple studies; and/or (e) were experimental studies, case-reports, case-series, reviews, systematic reviews or meta-analyses.

Here, HIV^+^ people were assigned to two groups according to their specific anti-*Toxoplasma* serological profiles: Those who (i) were test-positive for specific anti-*Toxoplasma* IgG antibody with low avidity, (ii) were test-positive for both specific anti-*Toxoplasma* IgM and IgG antibodies, (iii) had a seroconversion with a four to 16-fold increase in specific IgG titre over a period of 2–4 weeks, or (iv) were test-positive for circulating *Toxoplasma* antigen in serum, were assigned to the “acute *T. gondii* infection” (AT-) group [7]. Those who were test-positive for specific anti-*Toxoplasma* IgG serum antibody in the absence of IgM serum antibody [26] were assigned to the “latent *T. gondii* infection” (LT-) group.

### 2.2. Data Extraction and Assessment of Quality

We extracted the following variables from each study: first author’s name, country, city, year of publication, study design, number of participants, number of seropositive HIV^+^ people in each of the AT- and LT-groups, and the type of diagnostic method(s) used. Countries were grouped according to the WHO-defined regions [27], income per capita and the human development index [HDI]. In order to identify associated risk factors for toxoplasmosis in HIV^+^ people, we extracted data/information including: place of residence (urban/rural), gender (male or female), close contact with dogs or cats, contact with soil, and consumption of raw/undercooked meat, raw/unwashed vegetables and/or untreated drinking water. To evaluate the quality of the methodologies used in studies, we employed the Joanna Briggs Institute (JBI) prevalence critical appraisal tool [28].

### 2.3. Statistical Analysis

Analyses were performed using Stata statistical software (v.16 Stata Corp., College Station, TX, USA). For each individual study, prevalence was defined as the number of HIV^+^ individuals in either the AT- or LT-group, divided by the total number of HIV^+^ people studied. We used the variance of the study-specific prevalence estimates with the Freeman–Tukey double arcsine transformation prior to the pooling of data, employing the DerSimonian-Laird random-effects meta-analysis model to minimise the effect of studies with an extremely low or high reported prevalence [29,30,31]. The random-effects model [30] was selected in anticipation of significant variation in seroprevalence estimates among studies included here. Heterogeneity between studies was evaluated using Cochran’s Q test and the *I*^2^ index. *I*^2^ values of 25%, 50% and 75% represented low, medium and substantial heterogeneities, respectively [32]. To explore factors that might influence heterogeneity, several subgroup and meta-regression analyses were performed, considering the following study features: (i) WHO region, (ii) country, (iii) type of diagnostic method employed, (iv) study design, (v) income level in a country, and (vi) HDI level in a country. Moreover, to calculate the global number of seropositive HIV^+^ people in each of the AT- and LT-groups, we employed an established method [24] and used data from the WHO on the number of HIV^+^ people worldwide and in different countries/regions in 2019 [33]. Subsequently, we multiplied this number by the calculated percentages of HIV^+^ people in the AT- and LT-groups (95% CI) at the global and national levels. We did not undertake an assessment of publication bias, as this is not relevant for prevalence studies [34]. In all comparisons, a *p*-value of <0.05 was considered statistically significant.

## 3. Results

### 3.1. Study Characteristics

Of 4024 published scientific reports or papers, 150 and 65 studies met the inclusion criteria for analyses of the seroprevalences in the AT- and LT-groups, respectively (Figure S2). The 150 studies (155 data sets) surveyed 44,473 HIV^+^ people from 49 countries, whereas the 65 studies (65 datasets) investigated 17,705 HIV^+^ people from 32 countries. Relevant study characteristics are listed in Table S1. Most studies (*n* = 45) were from Africa, followed by the Western Pacific (*n* = 24) and the Eastern Mediterranean (*n* = 23). The lowest number of studies were from North America and the Caribbean (*n* = 12).

### 3.2. Global and Regional Seroprevalence Estimates in the LT-Group of HIV^+^ People, and Associated Socio-Demographic Aspects and Risk Factors

**Figure 1:**
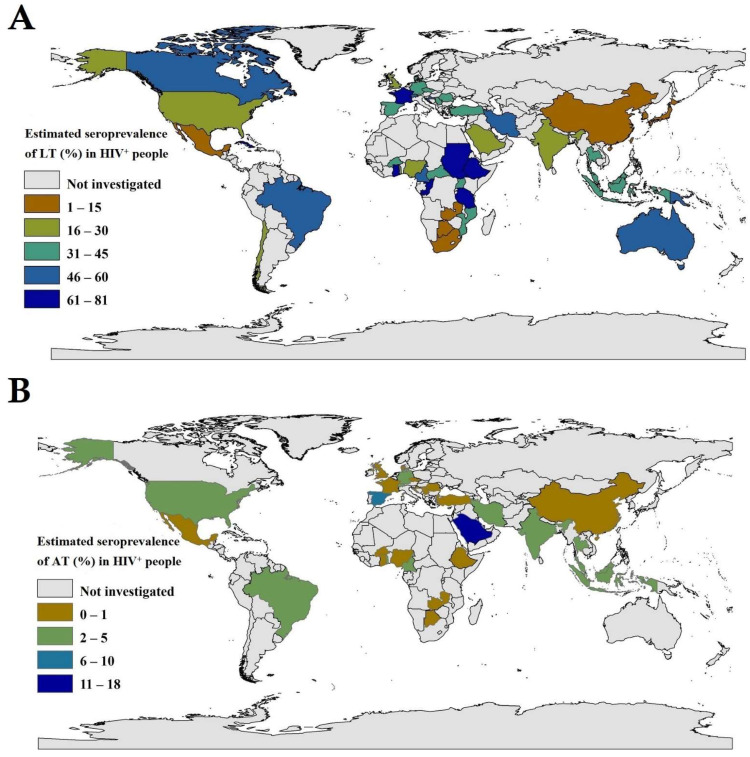
Seroprevalence estimates for latent or acute *T. gondii* infections ((**A**)—LT and (**B**)—AT) in HIV^+^ people in different countries around the world using the geographic information system (GIS).

Of the 44,473 HIV^+^ persons from 49 countries, 14,913 had specific anti-*T. gondii* IgG serum antibody profiles consistent with latent *T. gondii* infection (LT-group). Based on this evidence, we estimated an overall, pooled global prevalence of 37.4% (95% CI, 33.4–41.4) (Table 1; Figure 1A), with heterogeneity among studies (*I*^2^ = 98.7%, *p* < 0.001). For WHO regions, pooled seroprevalence rates (95% CI) were: 46.2% (37.7–54.7%) in Africa, 46.2% (29.6–62.6%) in South America, 45.8% (36.3–55.5%) in the Eastern Mediterranean region, 41.1% (33.0–49.4%) in Europe, 29.9% (22.0–38.3%) in South-East Asia, 25.5% (19.2–32.4%) in North America and 18.4% (12.4–25.3%) in the Western Pacific. For countries for which there were two or more eligible studies, Ethiopia (80.5%), Ghana (70.6%) and Cameroon (54.5%) in Africa; Iran (45.7%) in the Middle East; France (72.5%) and Austria (57.3%) in Europe; Brazil (48.8%) in South America; and Indonesia (38.5%), Thailand (37.5%) and Malaysia (36.1%) in East Asia exhibited some of the highest seroprevalences (Table 1 and Figure 1A). Extrapolating to the global population, we estimated that 14,174,600 (12,658,600–15,690,600) of HIV^+^ people had specific anti-*T. gondii* IgG serum antibody titres consistent with those expected of people with latent *T. gondii* infection (LT-group). These findings indicate clearly that countries in Africa have 11,873,400 (9,688,900–14,057,900) HIV^+^ people with latent *T. gondii* infection, equating to ~84% of HIV^+^ people worldwide. Details pertaining to estimated seroprevalences in distinct WHO-regions and countries are given in Table 1 and Figure 1A.

**Figure 2:**
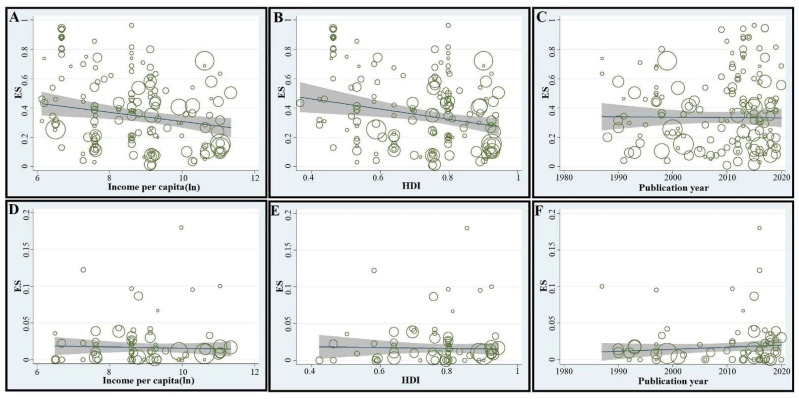
Meta-regression analyses of the seroprevalence rates of latent and acute *T. gondii* infections (LT and AT, respectively) in HIV^+^ people. For LT, there was a statistically significant downward trend in prevalence in countries with higher income levels or higher human development index (HDI), and an upward trend (statistically insignificant) from 1980 to 2020 (**A**–**C**, respectively). For AT, there was a downward trend (statistically insignificant) in seroprevalence in countries with higher income levels or higher HDIs, and an upward trend (statistically insignificant) from 1980 to 2020. ES: estimated seroprevalence (**D**–**F**).

In subgroup analyses, with respect to income level and HDI, the highest predicted seroprevalence for the LT-group was in low-income countries (58.2%, 46.2–69.8%) and the lowest in high-income countries (28.0%, 21.3–35.2%). The pooled prevalence in HIV^+^ people in the LT-group in countries with low, medium, high and very high HDIs were 51.9% (42.8–60.8%), 29.3% (20.9–38.4%), 38.0% (22.6–33.1%) and 27.6% (22.6–33.1%), respectively (Table 2). Meta-regression analyses revealed a significant decreasing trend in seroprevalence in countries with increasing levels of income per capita (*C* = −2.97 × 10^−6^; *p*-value = 0.004) and/or increasing HDI (*C* = −0.473; *p*-value < 0.001) (Figure 2A,B). Studies published after 2005 indicated slightly higher seroprevalences than before, although the increasing trend observed from 1980 to 2020 was non-significant upon meta-regression analysis (*C* = 0.0016; *p*-value = 0.46) (Table 2 and Figure 2C).

In subgroup analyses, according to the type of study, the highest and lowest seroprevalences were estimated in case-control investigations (40.4%; 30.7–50.6%) and retrospective cohort studies (26.5%; 17.1–37.2%), respectively. With respect to serological method used, subgroup analysis revealed the lowest (30.5%, 14.9–48.9%) and highest (57.2%, 47.0–67.1%) seroprevalences in studies that used the Sabin–Feldman (SFT) and immunofluorescence (IFAT) methods, respectively. The seroprevalence obtained in studies using ELISA (the most commonly used method) was 35.5% (31.1–40.1%). Further analyses and details are given in Table 2.

With respect to age, seroprevalences in the LT-group were 13.8% (11.8–15.7%), (39.5%, 38.7–40.4%), (46.3%, 44.9–47.7%) and (43.7%, 37.1–50.3%) in HIV^+^ people of < 20, 20–40, 40–60 and > 60 years of age, respectively (Table 3). With respect to the CD4+ lymphocyte counts, seroprevalences were 18.4% (16.8–20.0%), 33.8% (32.6–34.9%) and 21.9% (20.5–23.3%) in HIV^+^ people with CD4+ counts of <200, 200–500 and >500 per µL, respectively (Table 3).

Regarding risk factors, the findings showed that HIV^+^ people who consumed raw/undercooked meat (odds ratio [OR], 2.01; 95% CI, 1.19–3.9) and/or were in frequent contact with soil (OR, 3.01; 95% CI, 1.5–6.04) were more likely to be seropositive than those who consumed cooked meat and had limited contact with soil (Figures S3 and S4). Seroprevalence was significantly higher in HIV^+^ people who were older and had lower CD4+ cell counts. HIV^+^ people of 20–40 (OR, 1.63; 95% CI, 1.15–2.61), 40–60 (OR, 2.49; 95% CI, 1.62–3.82) and >60 (OR, 2.39; 95% CI, 1.56–3.66) years of age (Figures S5–S7) and those with CD4+ cell counts of 200–500 (OR, 1.71; 95% CI, 1.08–2.72) and <200 (OR, 1.04; 95% CI, 0.79–1.37) cells per µL were more likely to be seropositive (Figures S8 and S9). Other findings (Table 3 and Tables S10–S15) showed that females, people who lived in rural areas, consumed raw/unwashed vegetables or untreated water and/or were cat or dog owners were at a greater (but insignificant) risk of acquiring infection. No significant publication bias was seen, based on results achieved using the Egger’s test (Table 3).

### 3.3. Global and Regional Seroprevalence Estimates in the AT-Group of HIV^+^ People, and Associated Socio-Demographic Aspects

When the data from all 65 studies/datasets representing 32 countries were pooled, the global prevalence of acute *T. gondii* infection (AT) in HIV^+^ people was estimated at 1.3% (95% CI, 0.9–1.8%; 289 of 17,705). The heterogeneity between studies was significant (*I*^2^ = 79.1%, *p* < 0.001). The highest seroprevalences were indicated in South America (2.0%; 0.1–5.4%) and the Eastern Mediterranean region (1.8%; 0.7–3.3%), and the lowest prevalence in the European region (0.6%; 0.2–1.3%). The pooled seroprevalence rates in other WHO regions were: 1.6% (0.5–3.1%) in North America, 1.3% (0.9–1.8%) in South-East Asia, 1.2% (0.2–2.6%) in the Western Pacific and 0.9% (0.2–1.2%) in Africa (Table 4 and Figure 1B). We estimated that ~492,700 (341,100–682,200) HIV^+^ people worldwide were afflicted by acute *T. gondii* infection/toxoplasmosis. Our estimates demonstrated that Africa has the highest number of HIV^+^ people with acute infection (231,300; 51,400–308,400), accounting for ~47% of all such cases worldwide. Details pertaining to estimated seroprevalences in distinct WHO-regions and countries are given in Table 4 and Figure 1B. When the pooled seroprevalence was stratified according to income-level in a country, the highest prevalence estimates were in countries with lower-middle income levels (1.8%, 0.7–3.2%) and the lowest in those with low income-levels (0.4%, 0.0–1.9%). In relation to HDI, the highest prevalence rates were in countries with low HDIs (1.4%, 0.1–1.3%) and the lowest rates in countries with high HDIs (1.3%, 0.7–2.0%) (Table 2). Subsequent meta-regression analyses revealed an insignificant decreasing trend in prevalence in countries with increasing per capita incomes (C = −0.00082; *p*-value = 0.88) and HDIs (C = −0.0056; *p*-value = 0.92) in a country (Figure 2D,E). Considering study year, sub-group analysis showed higher prevalence rates from 2010 to 2020 than 1980 to 2009. This increasing trend was insignificant in meta-regression analysis (*C* = 0.0002; *p*-value = 0.7) (Figure 2F). Considering study-type, subgroup analysis showed the highest prevalence rates in case-control investigations (2.6%, 0.8–5.0%), followed by prospective cohort studies (2.6%, 0.8–5.0%), with the lowest seroprevalences estimated in retrospective cohort investigations (1.0%, 0.3–2.0%). Considering the serodiagnostic approach, subgroup analysis revealed that *Toxoplasma* seroprevalences estimated using IgG and IgM-based tests (1.2%, 0.7–1.8%) and seroconversion evaluation (1.2%, 0.8–1.7%) were almost the same. Additional subgroup analyses and results are given in Table 2.

## 4. Discussion

This investigation provides seroprevalence estimates to infer burdens of latent or acute *T. gondii* infection in HIV^+^ people at the global, regional and country levels, and sheds light on key socio-economic and immunological variables as well as risk factors for toxoplasmosis in this cohort of people.

The pooled seroprevalence rates estimated separately for latent (37.4; ~14.2 million people) and acute (1.3%; 0.5 million people) *T. gondii* infections in HIV^+^ people are similar to those reported (33.8% and 1.1%, respectively) for pregnant women worldwide [7,8]. The estimate for latent toxoplasmosis (37.4%) is slightly higher than the 35.8% reported previously for HIV^+^ people by Wang et al. [23], who used data from a total of 74 studies of 25,989 HIV^+^ people—i.e., 51% fewer studies and 42% fewer individuals than in the present investigation.

Here, the overall seroprevalence estimates for latent or acute *T. gondii* infection varied among regions, with the highest estimates in African and South-American countries and the lowest in the Western Pacific region, in agreement with previous global investigations of pregnant women [7,8]. The very high prevalence (up to ~ 84%) of latent infection in countries or regions of Africa (particularly sub-Sahara), Latin America and the Caribbean compared with other parts of the world (Table 1) likely relates to socioeconomic factors (income and HDI status) and climatic factors (high environmental temperature and humidity); the public health and sanitary situation; the prevalence of stray cats and the extent of environmental contamination with *T. gondii* oocysts (i.e., soil and water); cultural or culinary habits, particularly the consumption of semi-cooked or raw meat; and the infectivity/virulence of particular *T. gondii* genotypes present [7,11,35,36].

The present study showed that the key risk factors for latent *T. gondii* infection in HIV^+^ people are low CD4+ lymphocyte counts, age, frequent contact with soil, and the consumption of raw/undercooked meat, consistent with those proposed or documented in numerous previous publications and reviews [37,38,39,40,41]. Raw/undercooked meat is assumed to be a major source of *T. gondii* infection; however, as a risk factor, this depends on the kind of meat consumed and local culinary habits of meat preparation in a particular country or region; for example a report from China [42] indicated that the anti-*T gondii* seroprevalence in people who consumed beef or duck was higher than in than those who consumed pork, lamb and/or chicken. In European and American countries, lamb and pork are major dietary risk factors, whereas in the Middle East lamb is reported to be a key risk factor [37,40,43,44].

The increase in seroprevalence in HIV^+^ people with age is simply explained by an increased probability of infection with, or exposure to, *Toxoplasma* over time [19,36]. We expect that one of the most important risk factors for toxoplasmosis in HIV^+^ is a low CD4+ lymphocyte count. It is well documented that a decline in this count is a key reason for the reactivation of toxoplasmosis in HIV^+^ individuals with latent *T. gondii* infection, ultimately leading to severe or fatal disease [13,14]. Clearly, this scenario applies also to other immunodeficiencies caused by transplantation, malignant cancers and/or chemotherapy [17,45,46], where accidental or subclinical infections with opportunistic pathogens can have devastating consequences.

While the findings of the present study are insightful, some results need to be interpreted cautiously. First, we found significant heterogeneity between studies, which is a common issue in epidemiological studies such as this one, and could not be adequately explained by the subgroup and meta-regression analyses. The sources of heterogeneity likely relate to study characteristics, including differences in study design, season, geographical distribution, sample size and/or detection methods, including differences in the quality and performance of these methods. Second, the vast majority of studies in the literature have used serological methods to detect latent or acute *T. gondii* infection; however, serum antibody responses to *T. gondii* in immunodeficient individuals with HIV infection might be low in some or many cases, indicating or suggesting that the prevalence rates calculated here might actually be an under representation of the actual prevalence status globally and regionally. While bioassays (in mice or cats) are considered as the gold standard for the definitive diagnosis of toxoplasmosis [47], these assays are usually not used for diagnosis in humans. Moreover, DNA-based methods do not commonly complement routine diagnosis in humans. Thus, serodiagnosis is the mainstay, but variation in the performance of serological assays (including diagnostic specificity and sensitivity; reproducibility and repeatability of results) will influence prevalence estimates. Third, relatively few studies met the selection criteria for inclusion; many studies lacked critical data and/or information or were clinically focused on individual toxoplasmosis cases. Thus, the prevalence rates estimated here should be considered as apparent, not actual. In addition, we acknowledge that, despite undertaking a comprehensive systematic search, published studies and data sets are not available for many parts of the world.

## 5. Conclusions

In spite of some limitations, this is the most comprehensive review and meta-analysis of available data sets to estimate the seroprevalence of *T. gondii* infections in HIV^+^ people, and to identify associated risk factors. The inference that more than one third of all people with HIV infection likely have a latent *T. gondii* infection indicates that many of these individuals are at risk of developing ‘reactivated’ toxoplasmosis as a consequence of their immunodeficiency, and that a small percentage (~ 1%) are at risk of acquiring primary *Toxoplasma* infection. Current international guidelines [48] recommend that all HIV^+^ individuals should be tested for specific anti-*Toxoplasma* IgG serum antibodies immediately after being diagnosed as having HIV infection, and that particularly individuals with no detectable anti-*Toxoplasma* serum antibodies should be counselled about the risks of them acquiring *Toxoplasma* and/or other opportunistic infections. These guidelines also emphasise that *Toxoplasma*-seropositive patients with CD4+ counts of <100 cells/µL should receive prescribed, routine treatment with trimethoprim + sulfamethoxazole or atovaquone to prevent toxoplasmic encephalitis and *Pneumocystis jirovecii*-associated pneumonia. However, it is noteworthy that a 2017 systematic review of toxoplasmosis chemoprophylaxis failed to identify any studies from Africa [49], while a recent study from Mozambique highlights the aspects of this illness that are comparable to a neglected tropical disease at the treatment, prevention and policy levels [50]. The high seroprevalence rates of *T. gondii* infection in lesser developed countries and regions of the world, including those in sub-Saharan Africa, call for action by governments and health authorities to introduce the routine serological monitoring, counselling, care and/or prophylactic treatment measures needed to prevent severe toxoplasmosis from developing in highly susceptible people living with HIV infection.

## Figures and Tables

**Table 1:** Global, regional and national pooled seroprevalences of latent *T. gondii* infection (LT) in HIV^+^ people (results from 155 datasets performed in 48 countries).

WHO Region/Country	No. of Datasets	No. of HIV^+^ People Screened (Total)	No. of HIV^+^ People with LT	Pooled Sero- Prevalence (95% CI)	Estimated No. of HIV^+^ People with LT *
**Global**	**155**	**44,473**	**14,913**	**37.4 (33.4–41.4)**	**14,174,600 (12,658,600–15,690,600)**
**South Americas**	**9**	**2905**	**1270**	**46.2 (23.6–69.6)**	**877,800 (448,400–1,322,400)**
Brazil	8	650	1203	48.8 (23.5–74.5)	439,200 (211,500–670,500)
Chile	1	255	67	26.3 (21.0–32.1)	18,673 (14,910–22,791)
**African** **region**	**49**	**9504**	**3967**	**46.2 (37.7–54.70)**	**11,873,400 (9,688,900–14,057,900)**
Ethiopia	12	1778	1396	80.5 (66.3–91.6)	555,450 (457,740–632,040)
Nigeria	12	2149	709	30.3 (19.2–42.7)	575,700 (364,800–811,300)
Burkina Faso	5	2548	691	30.7 (25.5–36.2)	29,472 (24,480–34,752)
Cameroon	3	293	167	54.5 (37.5–71.1)	294,300 (202,500–383,940)
Ghana	2	519	365	70.6 (66.6–74.4)	232,980 (219,780–245,520)
Uganda	2	316	134	42.3 (36.9–47.8)	592,200 (516,600–669,200)
South Africa	2	407	62	13.5 (10.3–17.0)	1,039,000 (793,100–1,309,000)
Zambia	2	256	14	5.2 (2.7–8.4)	62,400 (32,400–100,800)
Mozambique	2	258	110	42.5 (36.5–47.7)	935,000 (803,000–1,049,400)
Tanzania	1	38	26	68.4 (51.3–82.5)	1,094,400 (820,800–1,320,000)
Canary island (Spain)	1	157	56	35.7 (28.2–43.7)	49,000 (39,480–61,180)
Botswana	1	46	3	6.5 (1.4–17.9)	24,050 (5180–66,230)
Togo	1	56	14	25.0 (14.4–38.4)	27,500 (15,840–42,240)
Congo	1	375	75	22.0 (19.1–26.4)	19,580 (16,999–23,496)
Congo (Democratic Republic of the)	1	38	28	73.6 (52.5–94.6)	331,200 (236,250–425,700)
Central African Republic	1	270	117	43.3 (37.3–49.5)	47,630 (41,030–54,450)
**Eastern Mediterranean**	**23**	**3151**	**1271**	**45.8 (36.3–55.5)**	**183,200 (145,200–222,000)**
Iran	19	2886	1148	45.7 (35.3–56.3)	27,877 (21,533–34,343)
Saudi Arabia	1	50	15	30.0 (17.9–44.6)	3,900 (2327–5798)
Bahrain	1	76	16	21.1 (12.5–31.9)	55 (32–83)
Morocco	1	95	59	62.1 (51.6–71.9)	13,041 (10,836–15,099)
Sudan	1	44	33	75.0 (59.7–86.8)	44,250 (35,223–51,212)
**European region**	**20**	**8786**	**4109**	**41.1 (33.0–49.4)**	**1,027,500 (825,000–1,235,000)**
Spain	4	1707	562	30.7 (9.4–57.7)	46,050 (14,100–86,550)
Turkey	2	788	352	45.2 (41.7–48.7)	6690 (6172–7207)
United Kingdom	2	609	164	26.9 (23.4–30.5)	27,330 (23,774–30,988)
France	2	1715	1237	72.5 (70.3–74.6)	130,500 (126,540–134,280)
Austria	2	659	377	57.3 (53.5–61.1)	5157 (4815–5499)
Romania	2	224	69	30.5 (24.6–36.8)	5490 (4428–6624)
Czech Republic	2	1302	20	40.0 (37.4–42.7)	1760 (1645–1879)
Croatia	1	166	86	51.8 (43.9–59.6)	829 (702–953)
Germany	1	183	64	35.0 (28.1–42.4)	30,450 (24,447–36,888)
Denmark	1	503	223	44.3 (39.9–48.8)	2746 (2474–3025)
Switzerland	1	715	360	50.3 (46.6–54.1)	10,060 (9320–10,820)
Serbia	1	288	127	44.1 (38.3–50.0)	1323 (1149–1500)
**South-East Asian** **Region**	**18**	**5232**	**1582**	**29.8 (22.0–38.3)**	**1,132,400 (836,000–1,455,400)**
India	10	2773	532	24.1 (16.8–32.2)	530,200 (369,600–708,400)
Indonesia	4	1131	447	38.5 (32.2–45.0)	246,400 (206,080–288,000)
Thailand	3	1328	603	37.5 (20.8–56.0)	180,000 (99,840–268,800)
**North America and the Caribbean**	**12**	**7202**	**1150**	**25.5 (19.2–32.4)**	**433,500 (326,400–550,800)**
USA	8	5862	889	18.3 (13.3–23.9)	201,300 (146,300–262,900)
Mexico	2	187	91	10.6 (8.8–12.6)	24,380 (20,240–25,980)
Canada	1	1074	14	48.7 (41.5–55.9)	30,681 (26,148–35,217)
Cuba	1	79	56	70.9 (59.6–80.6)	21,979 (18,476–24,986)
**Western Pacific** **Region**	**24**	**7630**	**1530**	**18.4 (12.4–25.3)**	**349,600 (235,600–480,700)**
China	10	3768	598	12.2 (5.5–20.9)	109,800 (49,500–188,100)
Malaysia	6	1507	511	36.1 (18.4–56.1)	31,407 (16,008–48,807)
Japan	4	680	67	9.9 (6.4–14.0)	2970 (1920–4200)
Taiwan	1	550	56	10.2 (7.8–13.0)	4896 (3744–6240)
South-Korea	1	173	7	4.0 (1.6–8.2)	1800 (720–3690)
Singapore	1	771	183	23.7 (20.8–26.9)	1872 (1643–2125)
Papua New Guinea	1	181	108	59.7 (52.1–66.9)	26,865 (23,445–30,105)

WHO regions are sorted according to prevalence rate, and countries according to the number of studies included. * Estimates of the numbers of HIV^+^ people in individual regions were obtained from a WHO report from 2019 [33].

**Table 2:** Seroprevalence estimates for latent or acute *T. gondii* infections (LT and AT, respectively) in HIV^+^ people according to a priori defined subgroups.

Variable/Subgroups	No. of Datasets		Total No. of HIV^+^ People Screened		No. of HIV^+^ People with LT	Pooled Sero-Prevalence % (95% CI)	No. of HIV^+^ People with AT	Pooled Sero-Prevalence % (95% CI)
	LT	AT	LT	AT				
**Income**								
Low	27	5	5950	973	2675	58.2 (46.2–69.8)	8	0.4 (0.0–1.9)
Lower middle	35	13	7168	3389	2350	34.5 (27.0–42.3)	72	1.8 (0.7–3.2)
Upper middle	55	28	13,980	5764	4682	36.0 (29.2–43.1)	105	1.4 (0.7–2.3)
High	38	20	17,375	7579	5206	28.0 (21.3–35.2)	104	1.2 (0.6–1.9)
**HDI**								
Low	42	8	7969	1307	3533	51.9 (42.8–60.8)	23	1.4 (0.1–3.7)
Medium	22	12	5556	3462	1554	29.3 (20.9–38.4)	64	1.5 (0.6–2.6)
High	47	25	13,405	6858	45269	38.0 (22.6–33.1)	109	1.3 (0.6–2.2)
Very high	44	21	17,543	6078	4557	27.6 (22.6–33.1)	93	1.3 (0.7–2.0)
**Type of study**								
Cross sectional	95	44	24,576	8739	7941	38.7 (33.6–44.0)	148	1.2 (0.7–1.8)
Case-control	32	10	5204	1895	2059	40.4 (30.7–50.6)	36	2.6 (0.8–5.0)
Prospective cohort	9	4	3534	1591	1151	36.6 (19.1–56.1)	34	2.1 (1.1–3.4)
Retrospective cohort	19	8	11,159	5480	3762	26.5 (17.1–37.2)	71	1.0 (0.3–1.0)
**Criteria for AT**								
IgG and IgM	na	50	na	9872	na	na	166	1.2 (0.7–1.8)
Seroconversion	na	12	na	6715	na	na	88	1.2 (0.8–1.7)
IgG avidity	na	2	na	961	na	na	28	1.7 (1.0–2.7)
Antigen detection	na	2	na	157	na	na	7	3.5 (1.0–7.2)
**Year**								
1980–2000	35	17	11,920	5894	4282	37.5 (29.4–46.0)	82	1.2 (0.6–1.9)
2000–2005	14	3	6366	1095	1823	26.2 (17.3–36.2)	6	0.5 (0.0–1.6)
2006–2010	17	4	2893	726	803	28.7 (15.7–43.8)	5	0.4 (0.0–1.2)
2011–2015	49	16	12,386	4212	4475	45.3 (37.4–53.3)	90	1.7 (0.7–3.2)
2016–2020	40	26	10,908	5688	3530	35.6 (27.8–43.9)	106	1.5 (0.7–2.5)
**Sample size**								
≤99	45	20	2987	1338	1269	41.5 (33.7–49.6)	55	2.9 (1.2–5.1)
100–300	65	27	11,526	4782	4807	43.1 (36.3–50.1)	77	1.0 (0.4–1.8)
301–500	22	10	8372	4016	2067	22.0 (14.5–30.7)	72	1.6 (1.0–2.4)
501–1000	16	7	11,132	4651	3530	29.9 (19.7–41.1)	57	0.9 (0.3–2.0)
>1000	7	2	10,456	2918	3241	29.8 (14.7–47.7)	28	1.0 (0.6–1.3)
**Risk of bias**								
Low	118	50	42,227	16,736	13,984	36.1 (31.6–40.6)	249	1.2 (0.8–1.6)
Moderate	37	16	2246	969	967	41.8 (33.4–41.4)	40	2.8 (1.0–5.4)
**Serological methods**								
ELISA	125	na	35,286	na	11,736	35.5 (31.1–40.1)	na	na
IFAT	9	na	2828	na	1486	57.2 (47.0–67.1)	na	na
LAT	5	na	658	na	395	61.9 (40.9–80.8)	na	na
MEIA	5	na	1631	na	321	30.1 (14.3–48.8)	na	na
SFT	6	na	3199	na	706	30.5 (14.9–48.9)	na	na
Other (MAT, CFT, ELFA or DAT)	5	na	871	na	269	40.2 (15.4–68.0)	na	na

Abbreviations: HDI: human development index; ELISA: enzyme-linked immunosorbent assay; IFAT: indirect immunofluorescence; LAT: latex agglutination; MEIA: microparticle enzyme immunoassay; SFT: Sabin-Feldman test; MAT: modified agglutination test; CFT: complement fixation test; ELFA: enzyme-linked fluorescence assay; DAT: direct agglutination test.

**Table 3:** Risk factors associated with HIV^+^ people inferred to have *Toxoplasma gondii* infection based on their serological profile.

Variables (Number of Studies)	No. of HIV^+^ People	No. Seropositive for *T. gondii*	Pooled Sero- Prevalence (95% CI)	OR (95% CI)	Heterogeneity *I*^2^ (%)	Publication Bias *p* Value |t|
**Gender (34)**					89.1	0.25
Female	5806	2106	35.16 (34.28, 36.03)	1		
Male	7826	2363	29.44 (28.60, 30.28)	0.78 (0.55–1.12)		
**Residence (9)**					36.9	0.44
Urban	1472	820	59.73 (57.61, 61.84)	1		
Rural	283	166	67.29 (63.16, 71.42)	1.45 (0.76–2.75)		
**Close contact with dog (3)**					90.1	0.98
No	531	141	17.47 (14.89, 20.05)	1		
Yes	235	56	16.36 (12.46, 20.26)	2.69 (0.55–13.18)		
**Close contact with cats (15)**					84.0	0.31
No	1753	919	76.52 (74.94, 78.10)	1		
Yes	1039	611	75.39 (73.13, 77.64)	1.79 (0.91–3.50)		
**Contact with soil (5)**					67.2	0.5
No	442	219	46.84 (43.18, 50.49)	1		
Yes	316	236	83.92 (80.26, 87.58)	3.01 (1.50–6.04)		
**Consumption of raw meat (15)**					78.5	0.88
No	1626	892	68.09 (66.65, 69.53)	1		
Yes	1016	699	85.28 (83.54, 87.02)	2.01 (1.19–3.39)		
**Consumption of raw/unwashed vegetable (8)**					13.7	0.26
No	493	411	88.79 (86.14, 91.43)	1		
Yes	756	653	95.28 (93.82, 96.74)	1.04 (0.68–1.6)		
**Drinking untreated water (5)**					45.5	0.19
No	835	612	82.19 (79.95, 84.44)	1		
Yes	298	207	83.64 (80.24, 87.03)	1.19 (0.67–2.11)		
**Number of CD4+ cells (29)**						
≥ 500	1733	440	18.48 (16.88, 20.09)	1		
200–500	3625	1201	33.82 (32.68, 34.97)	1.71 (1.08–2.72)	50.9	0.71
< 200	2511	700	21.97 (20.57, 23.36)	1.04 (0.79–1.37)	77.2	0.51
**Age (36)**						
< 20	1064	181	13.81 (11.86, 15.77)	1		
20–40	6824	2393	39.59 (38.70, 40.47)	1.63 (1.15–2.61)	42.3	0.06
40–60	2968	1295	46.33 (44.93, 47.74)	2.49 (1.62–3.82)	54.2	0.09
>60	181	75	43.78 (37.19, 50.36)	2.39 (1.56–3.66)	0	0.48

**Table 4:** Global, regional and national pooled seroprevalence rates for acute *Toxoplasma gondii* infection (AT) in HIV^+^ people (results from 65 studies performed in 31 countries).

WHO Regions/Country	No. of Datasets	No. of HIV^+^ People Screened (Total)	No. of HIV^+^ People Estimated to Have AT	Pooled Prevalence (95% CI)	Estimated No. of HIV^+^ People with AT *
**Global**	**65**	**17,705**	**289**	**1.3 (0.9–1.8)**	**492,700 (341,100–682,200)**
**South Americas**	**4**	**863**	**20**	**2.0 (0.1–5.4)**	**38,000 (1900–102,600)**
Brazil	4	863	20	2.0 (0.1–5.4)	38,000 (1900–102,600)
**African** **region**	**15**	**2505**	**32**	**0.9 (0.2–1.2)**	**231,300 (51,400–308,400)**
Burkina Faso	2	497	0	0.1 (0.0–0.4)	96 (0–384)
Cameroon	2	223	14	5.4 (2.7–8.9)	29,160 (14,580–48,060)
Ghana	2	519	1	0.1 (0.0–0.8)	330 (0–2640)
South Africa	2	407	7	1.4 (0.4–2.9)	107,800 (30,800–223,300)
Ethiopia	1	150	0	1.1 (0.2–2.4)	7590 (1380–16,560)
Nigeria	1	111	1	0.9 (0.1–4.9)	17,100 (1900–93,100)
Zambia	1	69	0	0.1 (0.0–5.2)	1200 (0–62,400)
Canary island (Spain)	1	157	1	0.6 (0.0–3.5)	840 (0–4900)
Botswana	1	46	0	0.1 (0.0–7.7)	370 (0–28,490)
Togo	1	56	2	3.6 (0.4–12.3)	3960 (440–13,530)
**Eastern Mediterranean**	**15**	**2125**	**51**	**1.8 (0.7–3.3)**	**7200 (2800–13,200)**
Iran	13	1999	42	1.5 (0.6–2.7)	915 (366–1647)
Saudi Arabia	1	50	9	18.0 (8.6–31.4)	2340 (1118–4082)
Bahrain	1	76	0	0.1 (0.0–4.7)	2 (0–12.2)
**European region**	**15**	**6447**	**67**	**0.6 (0.2–1.3)**	**15,000 (5000–32,500)**
Turkey	2	788	0	0.1 (0.0–0.2)	15 (0–29.6)
France	2	1715	14	0.3 (0.0–0.7)	540 (0–1260)
Romania	2	224	2	0.1 (0.0–1.0)	18 (0–180)
Czech Republic	2	1302	14	0.8 (0.3–1.4)	35 (13–61)
Spain	1	63	6	9.5 (3.6–19.6)	14,250 (5400–29,400)
United Kingdom	1	500	7	1.4 (0.6–2.9)	1422 (609–2946)
Croatia	1	166	2	1.2 (0.1–4.3)	19 (2–69)
Germany	1	183	6	3.3 (1.2–7.0)	2871 (1044–6090)
Denmark	1	503	4	0.8 (0.2–2.0)	49 (12–124)
Switzerland	1	715	12	1.7 (0.9–2.9)	340 (180–580)
Serbia	1	288	0	0.1 (0.0–1.3)	3 (0–39)
**North America and the Caribbean**	**5**	**1729**	**28**	**1.6 (0.5–3.1)**	**27,200 (8500–52,700)**
USA	4	1637	27	1.7 (0.5–3.6)	18,700 (5500–39,600)
Mexico	1	92	1	1.1 (0.1–5.9)	2530 (230–13,570)
**South-East Asian Region**	**9**	**3605**	**85**	**1.3 (0.9–1.8)**	**49,400 (34,200–68,400)**
India	5	1730	27	1.6 (0.4–3.4)	35,200 (8800–74,800)
Indonesia	3	737	29	3.9 (2.5–5.4)	24,960 (16,000–34,560)
Thailand	2	1138	29	1.5 (0.8–2.3)	7200 (3840–11,040)
**Western Pacific Region**	**3**	**441**	**6**	**1.2 (0.2–2.6)**	**22,800 (3800–49,400)**
Malaysia	2	182	3	1.5 (0.1–4.1)	1305 (87–3567)
China	1	259	3	1.2 (0.2–3.3)	10,800 (1800–29,700)

WHO regions are sorted according to prevalence rate, and countries according to the number of studies included. * Estimates of the numbers of HIV^+^ people in individual regions were obtained from a WHO report from 2019 [33].

## Data Availability

All data are available in main manuscript and supplementary files. Further data would be provided by the first corresponding author as/if requested.

## Supplementary Materials

The following are available online at https://www.mdpi.com/article/10.3390/microorganisms9102034/s1, Figure S1: Search strategy in databases, Figure S2: Flow diagram of the search strategy and study selection process, Figure S3: Forest plots of pooled odds ratio (OR) for latent *T. gondii* infection in HIV^+^ people with respect to consumption of raw/under-cooked meat. HIV^+^ people who did not consume raw/under-cooked meat were considered as the reference category to estimate OR, Figure S4: Forest plots of pooled odds ratio (OR) for latent *T. gondii* infection in HIV^+^ people with respect to contact with soil. HIV^+^ people without contact with soil were considered as the reference category to estimate OR, Figure S5: Forest plots of pooled odds ratio (OR) for latent *T. gondii* infection in HIV^+^ people with respect to ages of 20–40 years compared to ages <20, Figure S6: Forest plots of pooled odds ratio (OR) for latent *T. gondii* infection in HIV^+^ people with respect to the ages of 40–60 years compared to ages <20, Figure S7: Forest plots of pooled odds ratio (OR) for latent *T. gondii* infection in HIV^+^ people with respect to ages >60 years compared to ages <20, Figure S8: Forest plots of pooled odds ratio (OR) for latent *T. gondii* infection in HIV^+^ people with respect to age and number of CD4+ cells 200–500 compared to age and number of CD4+ cells >500, Figure S9: Forest plots of pooled odds ratio (OR) for latent *T. gondii* infection in HIV^+^ people with respect to age and number of CD4+ <200 compared to age and number of CD4+ >500, Figure S10: Forest plots of pooled odds ratio (OR) for latent *T. gondii* infection in HIV^+^ people with respect to gender (male and female). Female gender was considered as the reference category to estimate OR, Figure S11: Forest plots of pooled odds ratio (OR) for latent *T. gondii* infection in HIV^+^ people with respect to type of residence (rural and urban). Living in an urban area was considered as the reference category to estimate OR, Figure S12: Forest plots of pooled odds ratio (OR) for latent *T. gondii* infection in HIV^+^ people with respect to cat ownership. HIV^+^ people who were not cat owners were considered the reference category to estimate OR, Figure S13: Forest plots of pooled odds ratio (OR) for latent *T. gondii* infection in HIV^+^ people with respect to dog ownership. HIV^+^ people who were not dog owners were considered the reference category to estimate OR, Figure S14: Forest plots of pooled odds ratio (OR) for latent *T. gondii* infection in HIV^+^ people with respect to consumption of raw/unwashed vegetables. HIV^+^ people who did not consume raw/unwashed vegetables were considered as the reference category to estimate OR, Figure S15: Forest plots of pooled odds ratio (OR) for latent *T. gondii* infection in HIV^+^ people with respect to drinking untreated water. HIV^+^ people who drank treated water were considered as the reference category to estimate OR, Table S1: Main features of studies included on the seroprevalences of latent and acute *Toxoplasma gondii* infection (LT and AT, respectively) in HIV^+^ people worldwide.
