# Supplementary material for: Seroprevalence Estimates of Latent and Acute Toxoplasma Infections in HIV+ People—Call for Action in Underprivileged Communities
Source: Microorganisms. 2021 Sep 26;9(10):2034. doi: 10.3390/microorganisms9102034 (PMC8537859; doi:10.3390/microorganisms9102034)
Supplement: Supplementary file 1 [file microorganisms-09-02034-s001.zip › Supplementary Figures.pdf]

PubMed = 1020

("Toxoplasma gondii" OR "Toxoplasma infection" OR toxoplasmosis) AND ("human immunodeficiency viruses" OR HIV OR AIDS OR "acquired immunodeficiency syndrome") AND (prevalence OR seroprevalence OR seroepidemiology OR coinfection)

Scopus = 770

TITLE-ABS-KEY (("Toxoplasma gondii" OR "Toxoplasma infection" OR toxoplasmosis) AND ("human immunodeficiency viruses" OR HIV OR aids OR "acquired immunodeficiency syndrome") AND (prevalence OR seroprevalence OR seroepidemiology OR coinfection)).

Figure S1. Search strategy in databases

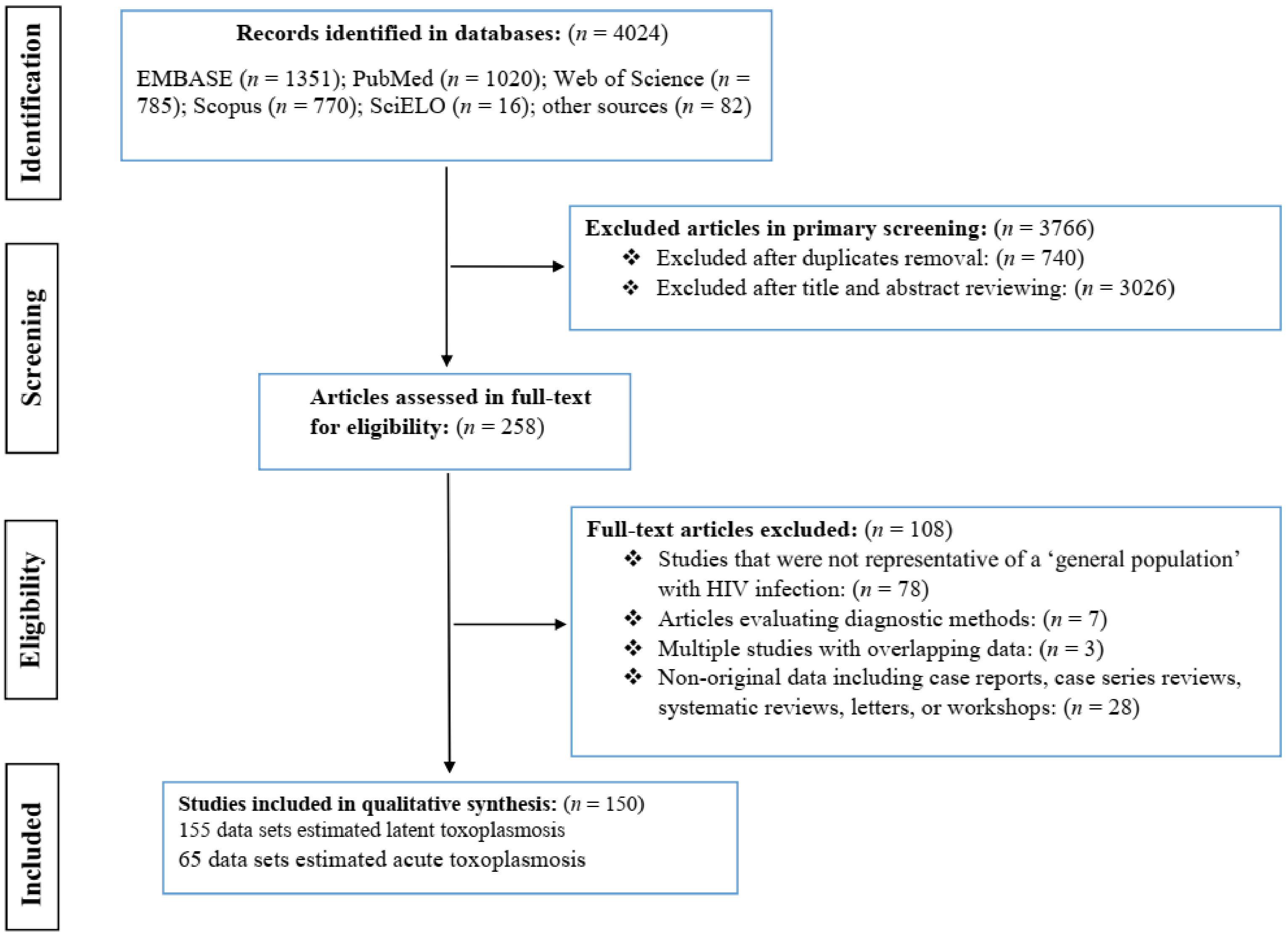

Figure S2. Flow diagram of the search strategy and study selection process.

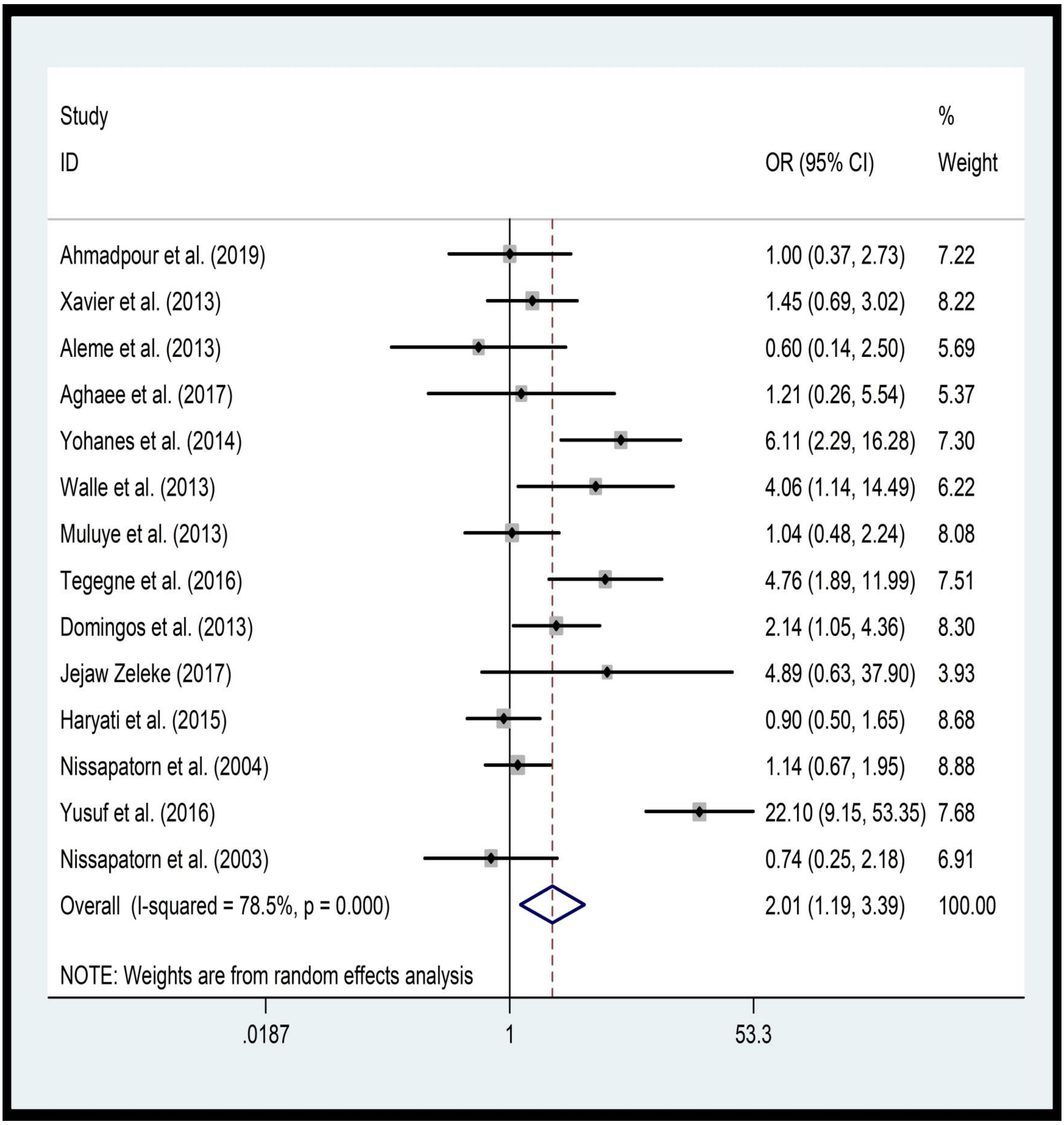

Figure S3. Forest plots of pooled odds ratio (OR) for latent *T. gondii* infection in HIV^+ people with respect to consumption of raw/under-cooked meat. HIV^+ people who did not consume raw/under-cooked meat were considered as the reference category to estimate OR.

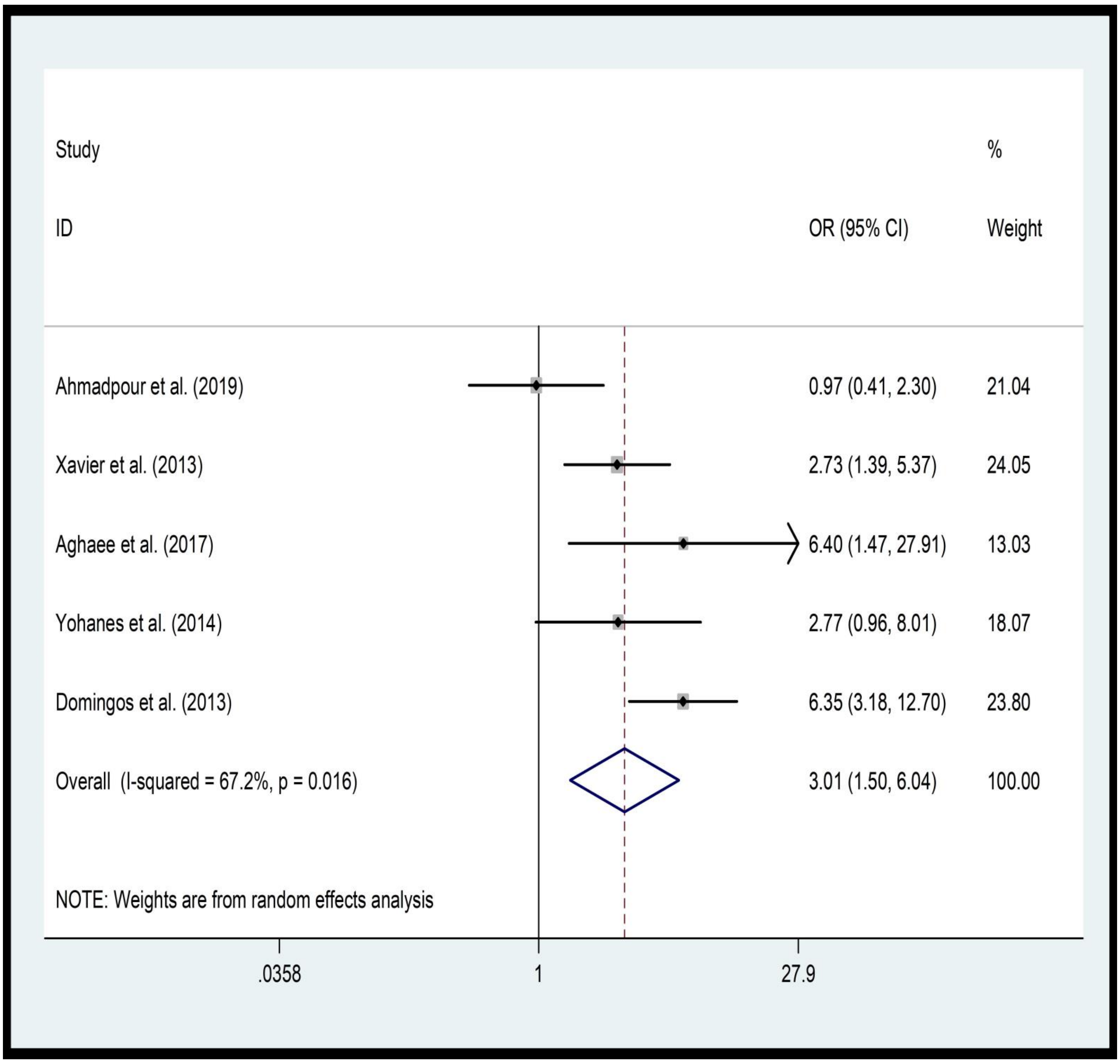

Figure S4. Forest plots of pooled odds ratio (OR) for latent *T. gondii* infection in HIV^+ people with respect to contact with soil. HIV^+ people without contact with soil were considered as the reference category to estimate OR.

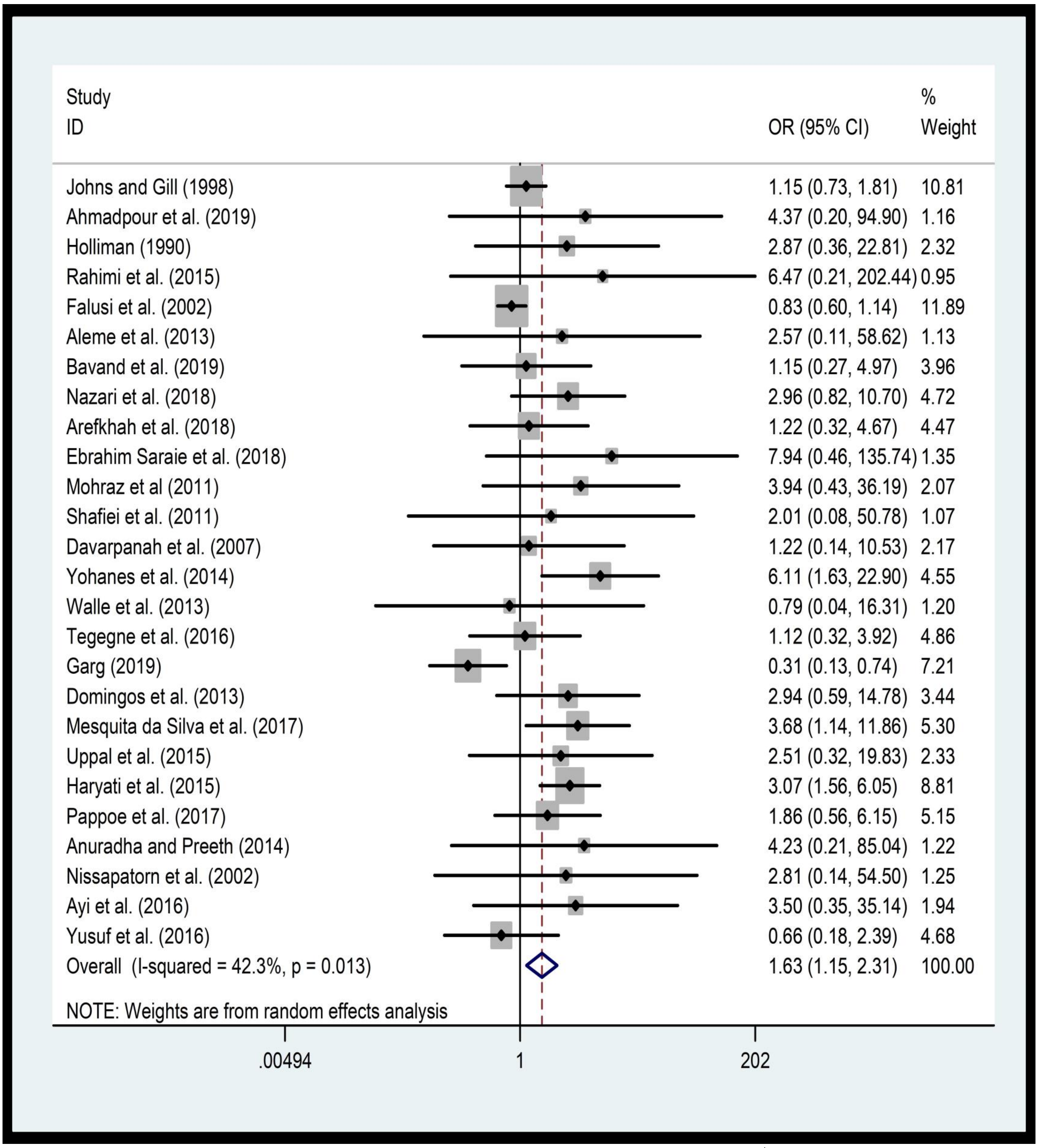

Figure S5. Forest plots of pooled odds ratio (OR) for latent *T. gondii* infection in HIV^+ people with respect to ages of 20-40 years compared to ages <20.

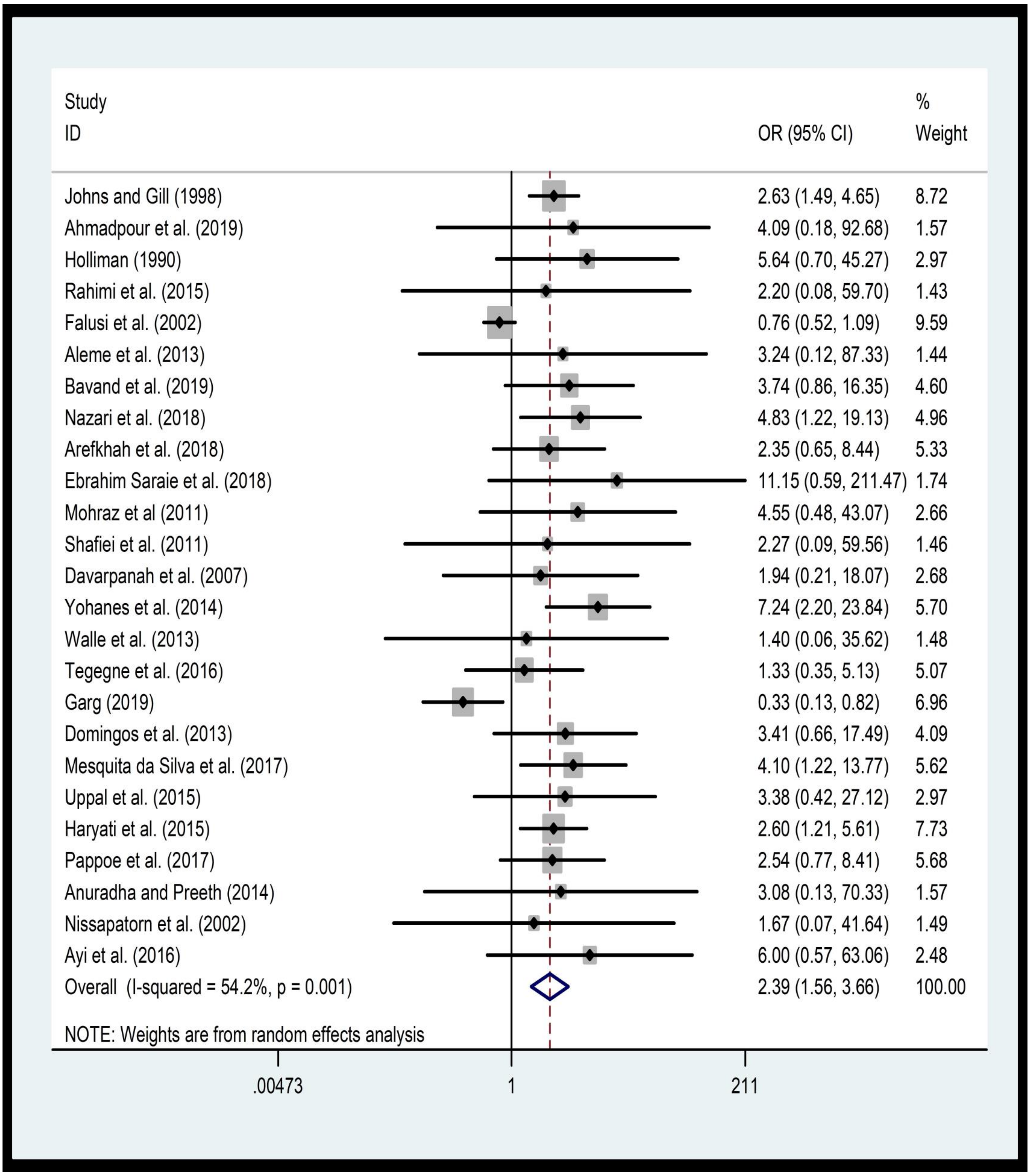

Figure S6. Forest plots of pooled odds ratio (OR) for latent *T. gondii* infection in HIV^+ people with respect to ages of 40-60 years compared to ages <20.

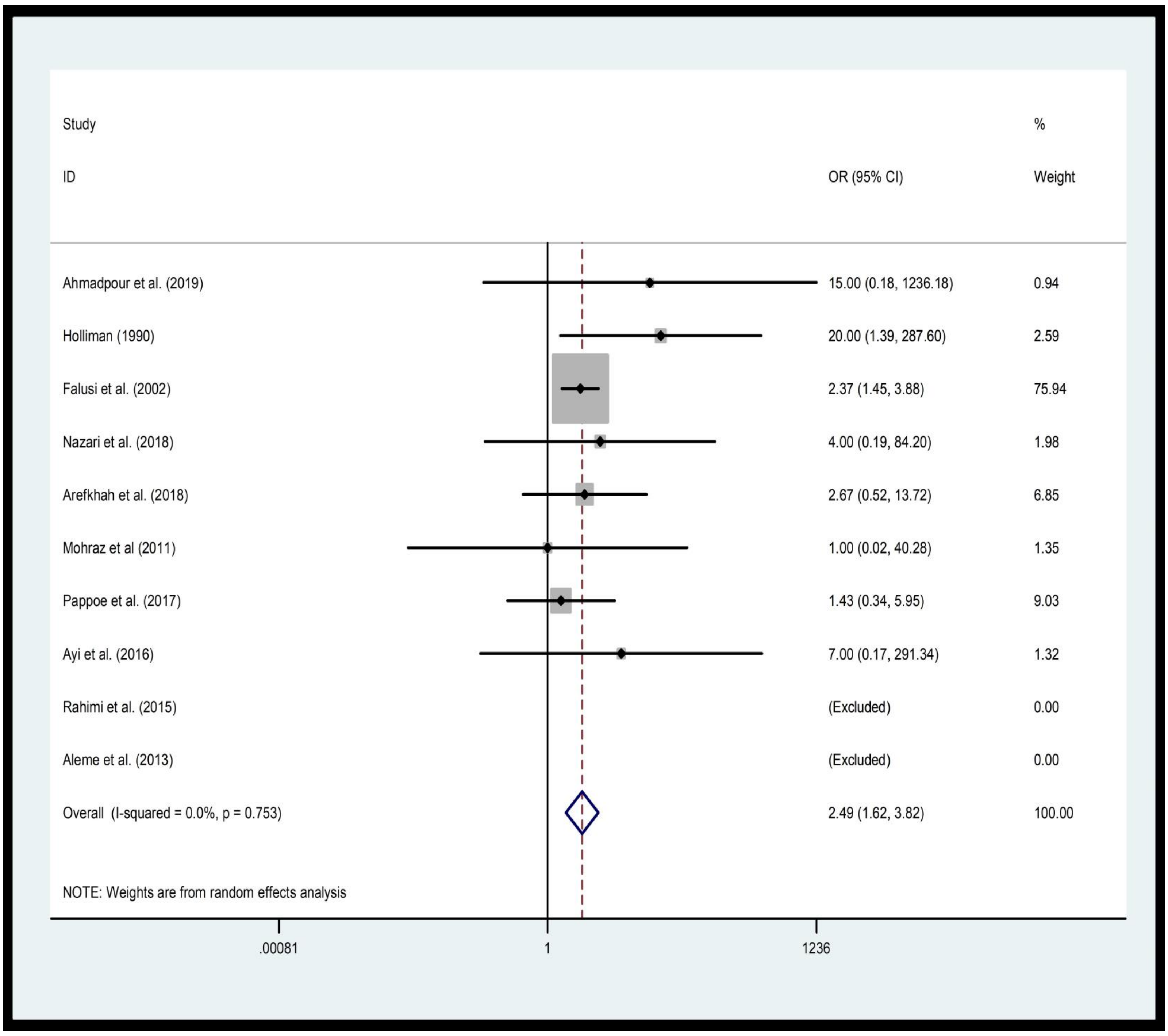

Figure S7. Forest plots of pooled odds ratio (OR) for latent *T. gondii* infection in HIV^+ people with respect to ages >60 years compared to ages <20.

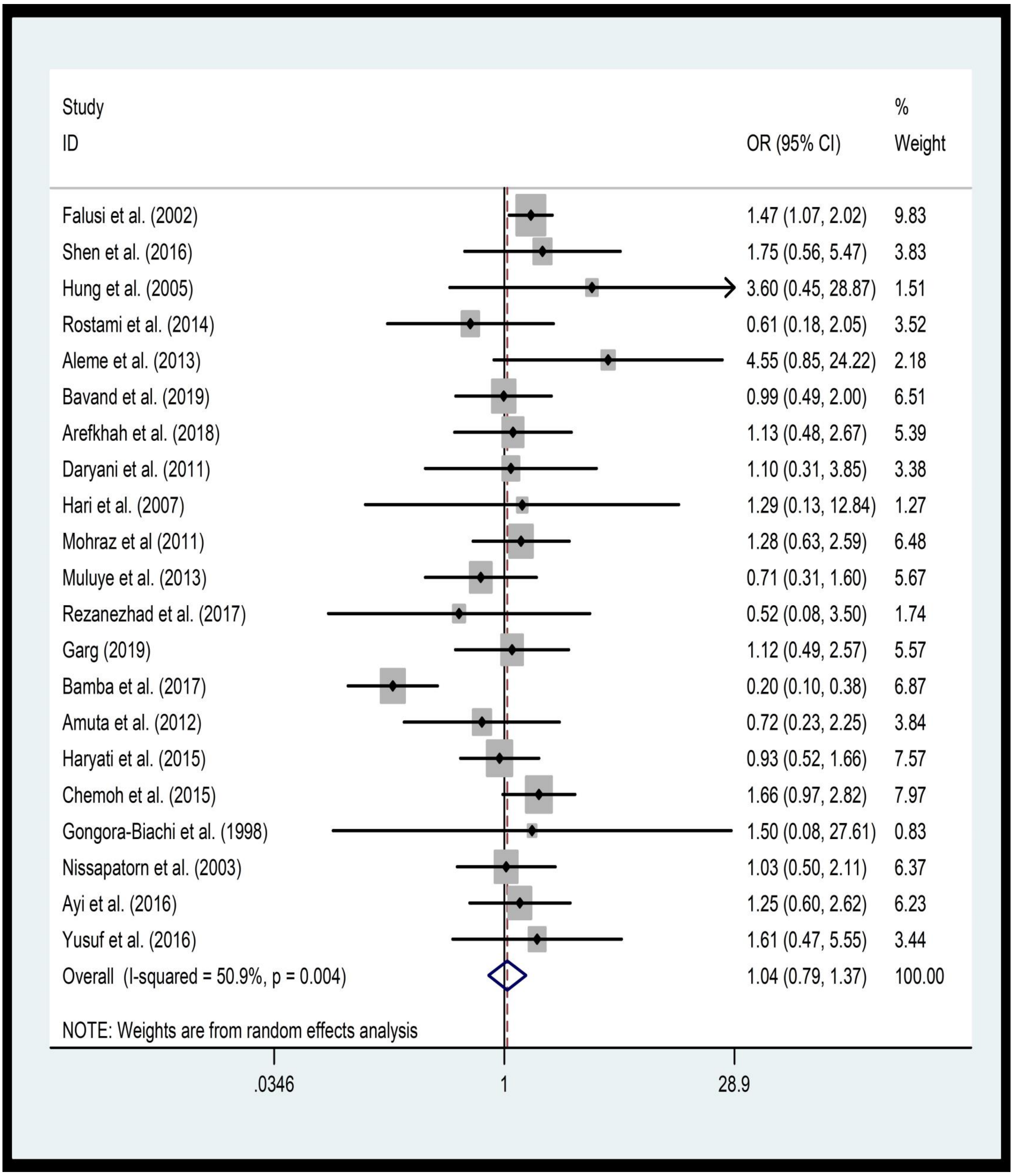

Figure S8. Forest plots of pooled odds ratio (OR) for latent *T. gondii* infection in HIV^+ people with respect to age and number of CD4+ cells 200-500 compared to age and number of CD4+ cells >500.

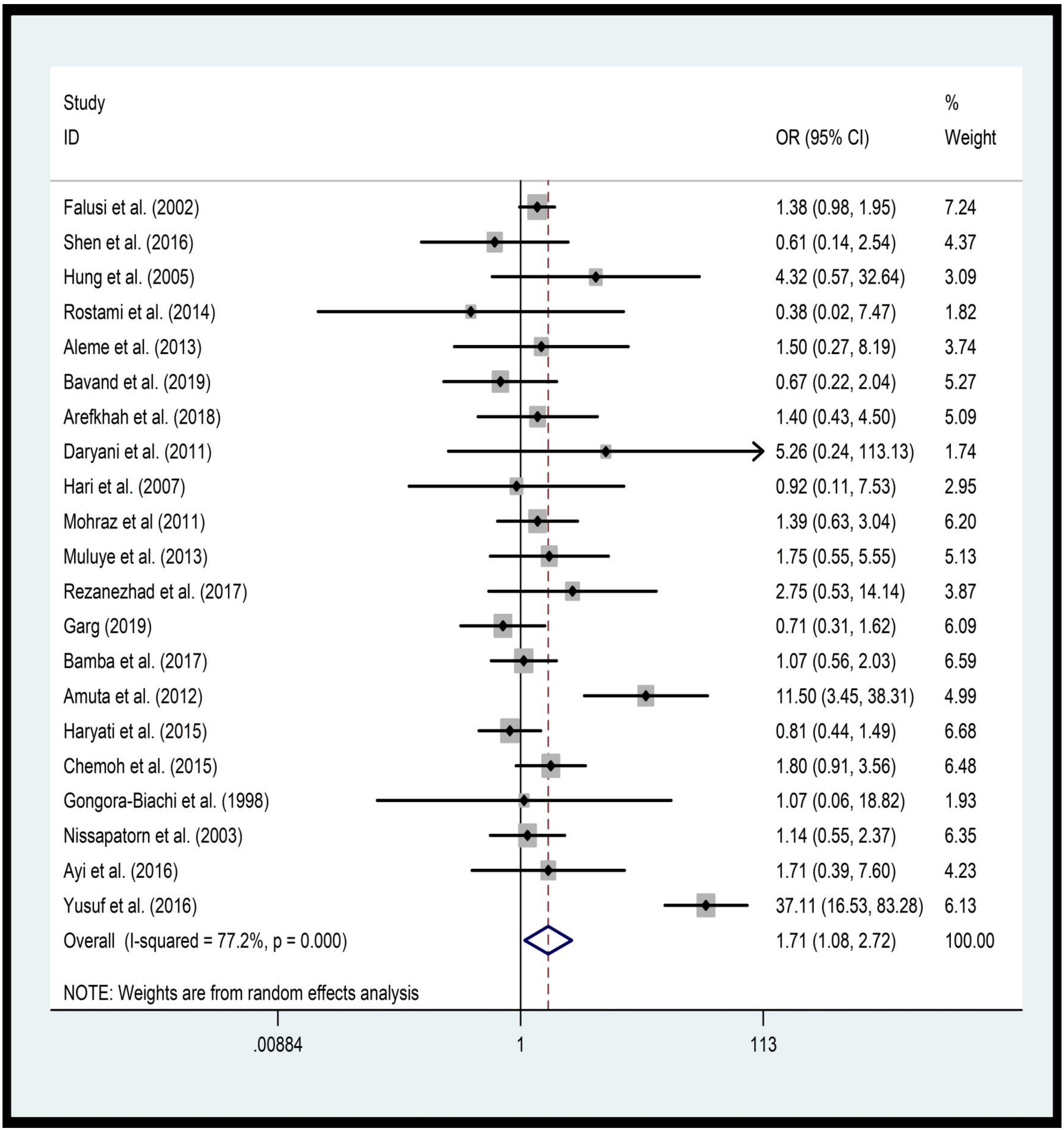

Figure S9. Forest plots of pooled odds ratio (OR) for latent *T. gondii* infection in HIV^+ people with respect to age and number of CD4+ <200 compared to age and number of CD4+ >500.

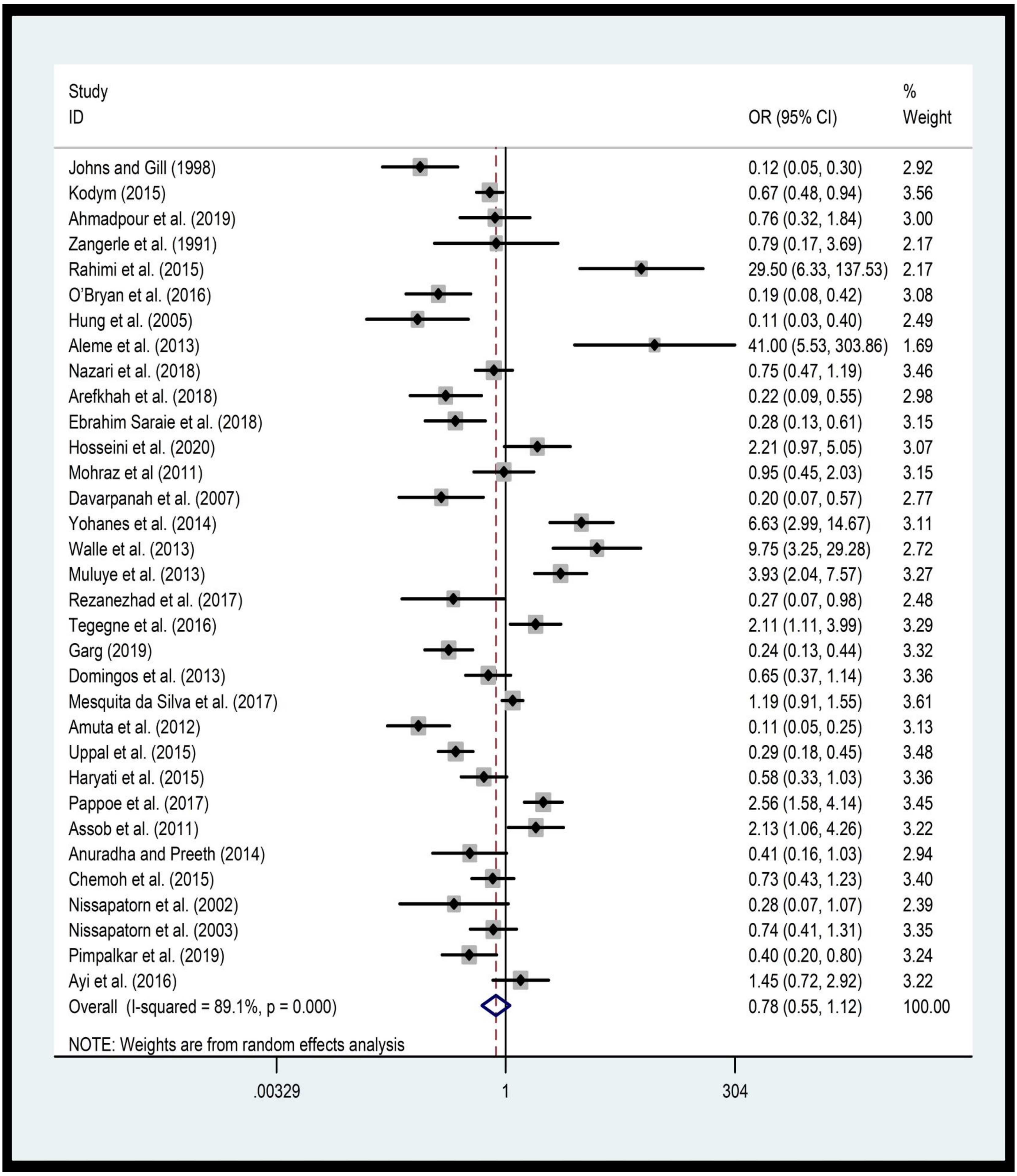

Figure S10. Forest plots of pooled odds ratio (OR) for latent *T. gondii* infection in HIV^+ people with respect to gender (male and female). Female gender was considered as the reference category to estimate OR.

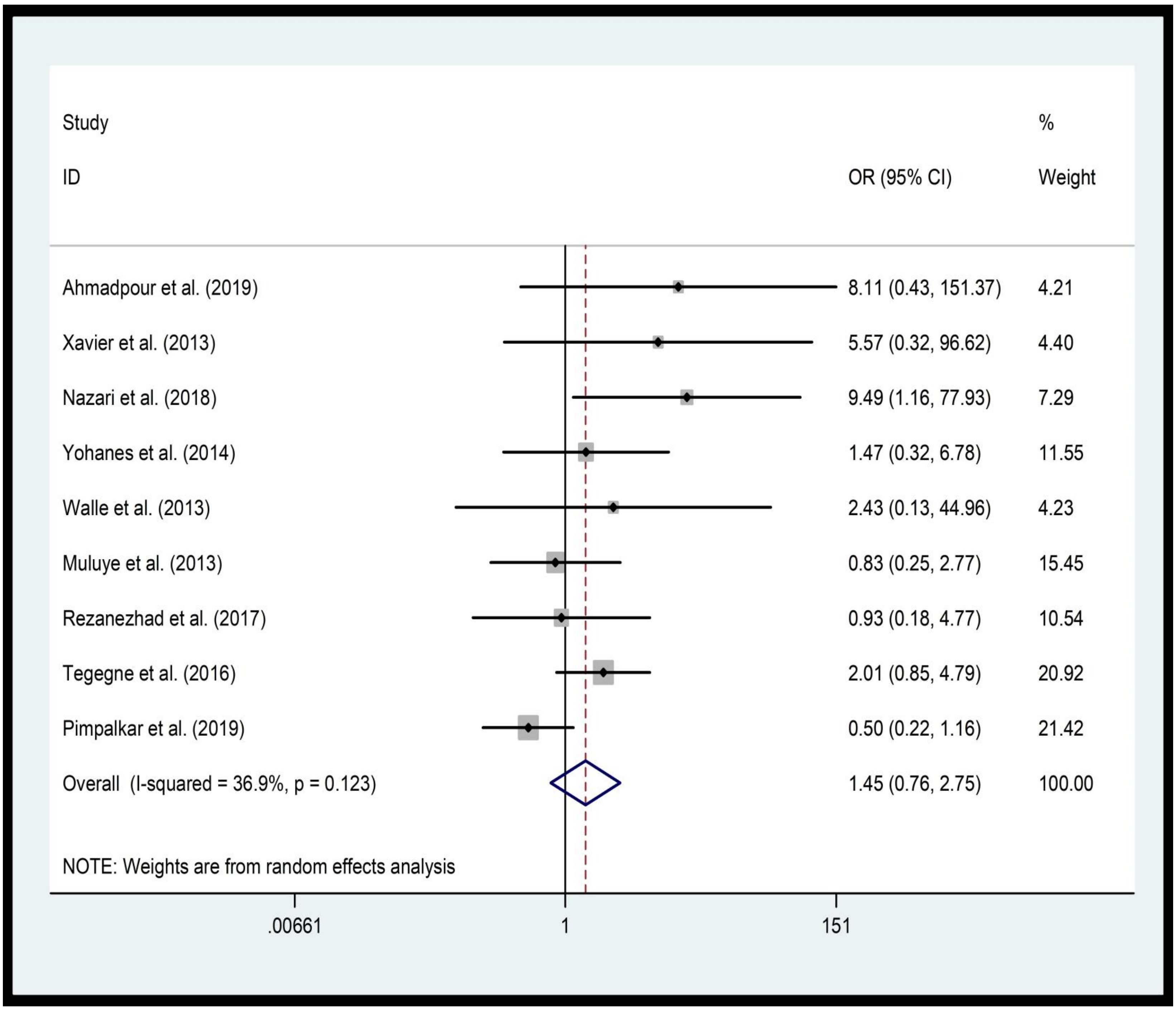

Figure S11. Forest plots of pooled odds ratio (OR) for latent *T. gondii* infection in HIV$^+$ people with respect to type of residence (rural and urban). Living in an urban area was considered as the reference category to estimate OR.

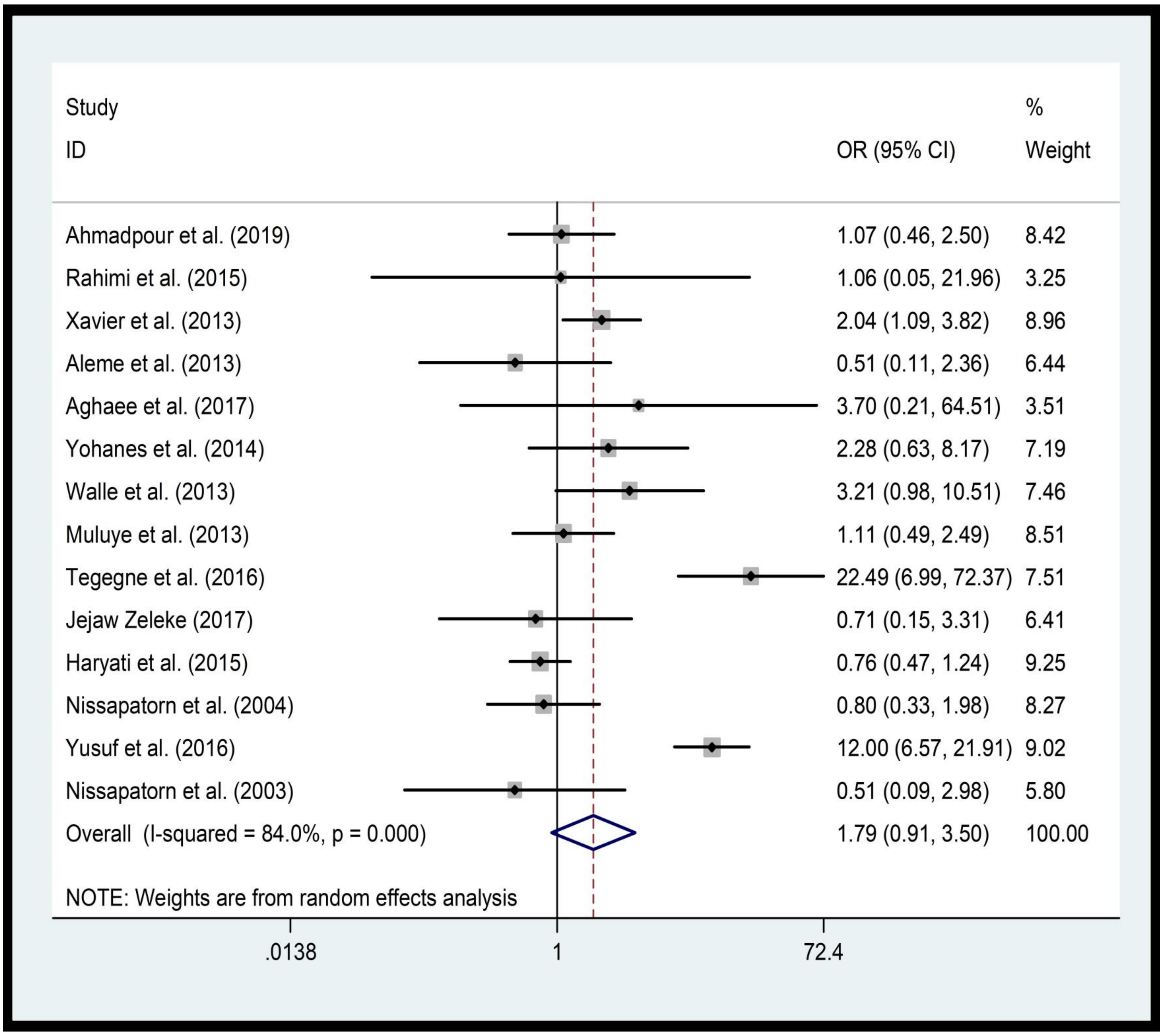

Figure S12. Forest plots of pooled odds ratio (OR) for latent *T. gondii* infection in HIV^+ people with respect to cat ownership. people living with HIV who were not cat owners were considered the reference category to estimate OR.

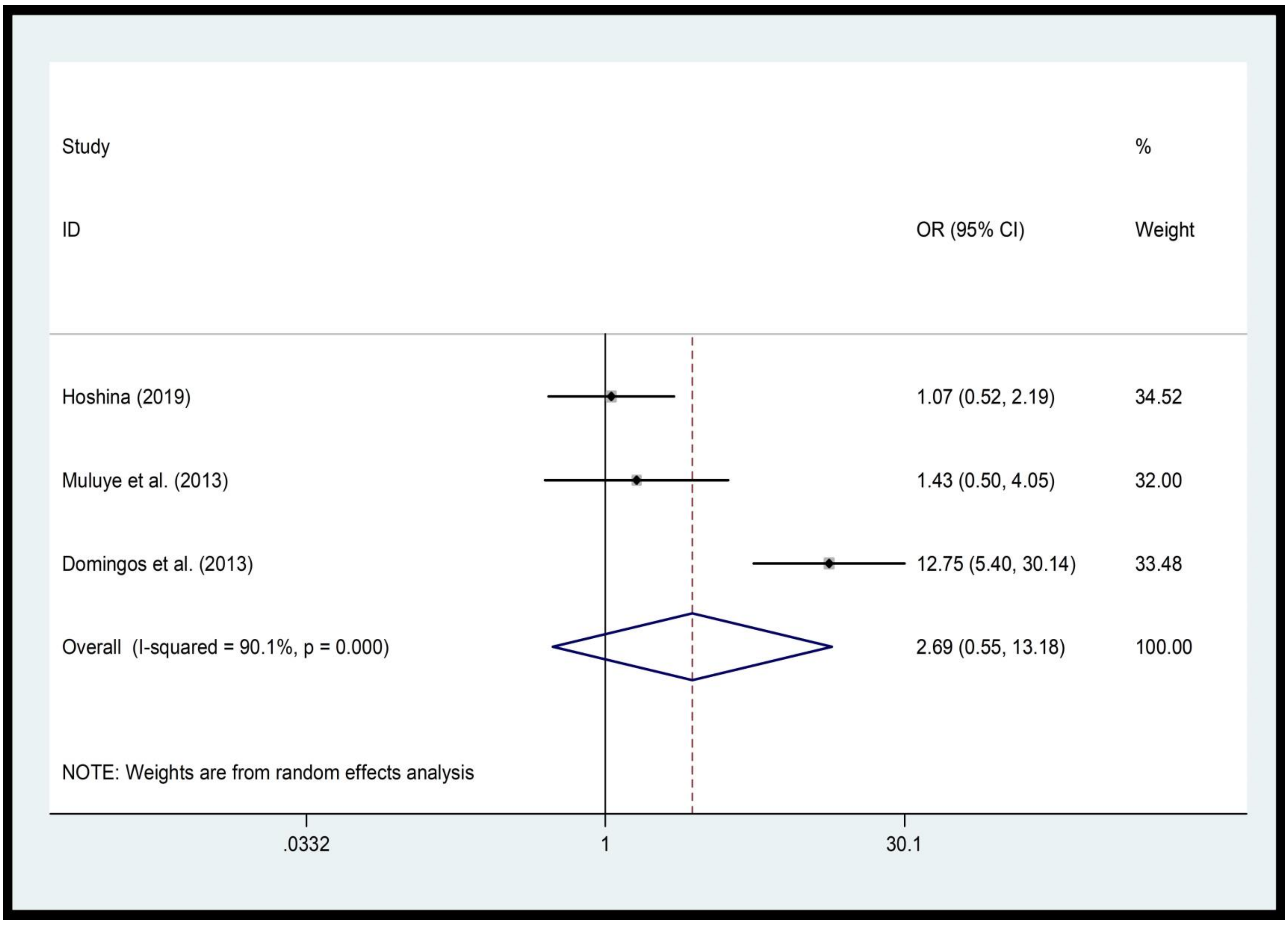

Figure S13. Forest plots of pooled odds ratio (OR) for latent *T. gondii* infection in HIV^+ people with respect to dog ownership. HIV^+ people who were not dog owners were considered the reference category to estimate OR.

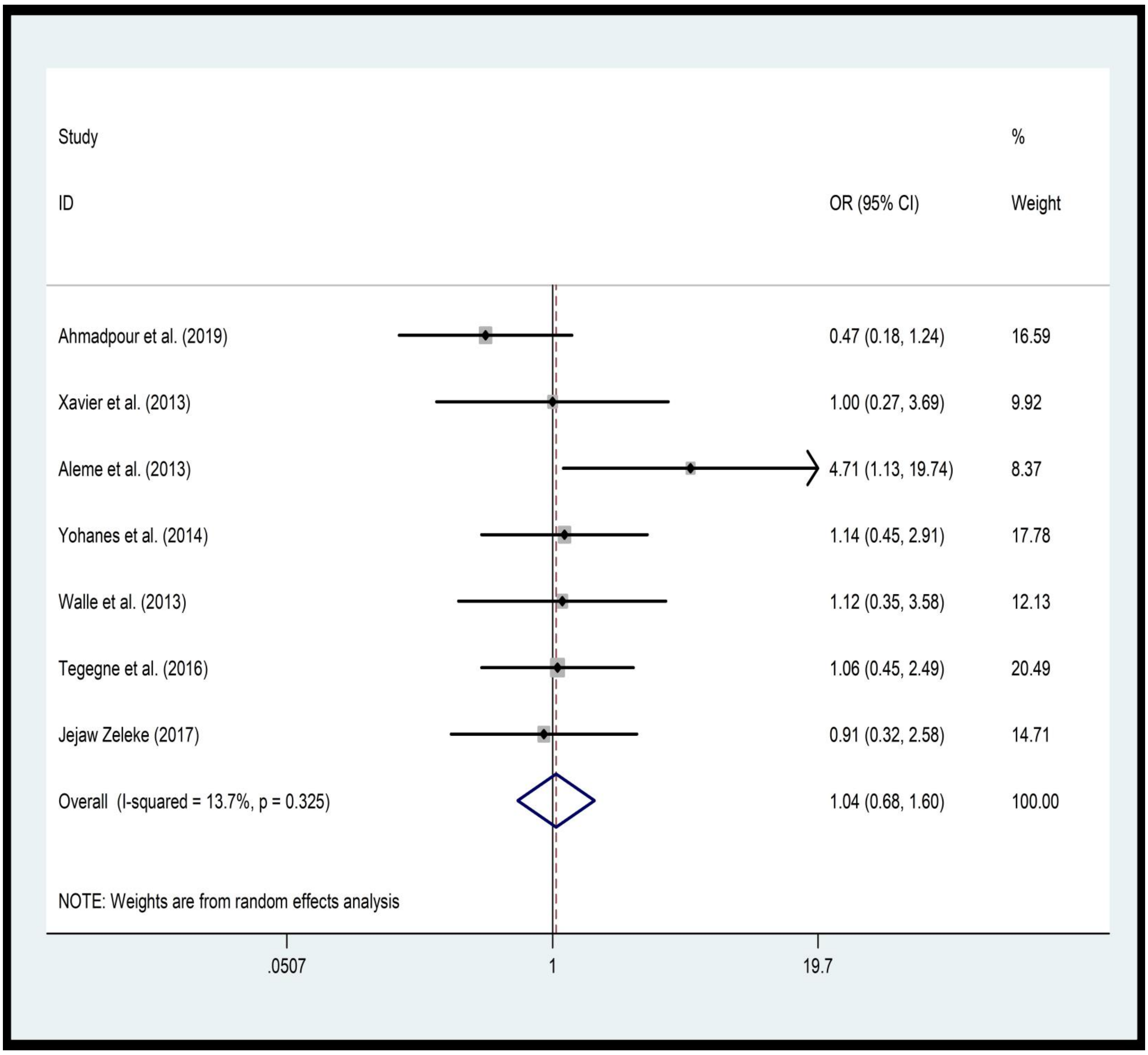

Figure S14. Forest plots of pooled odds ratio (OR) for latent *T. gondii* infection in HIV$^+$ people with respect to consumption of raw/unwashed vegetables. HIV$^+$ people who did not consume raw/unwashed vegetables were considered as the reference category to estimate OR.

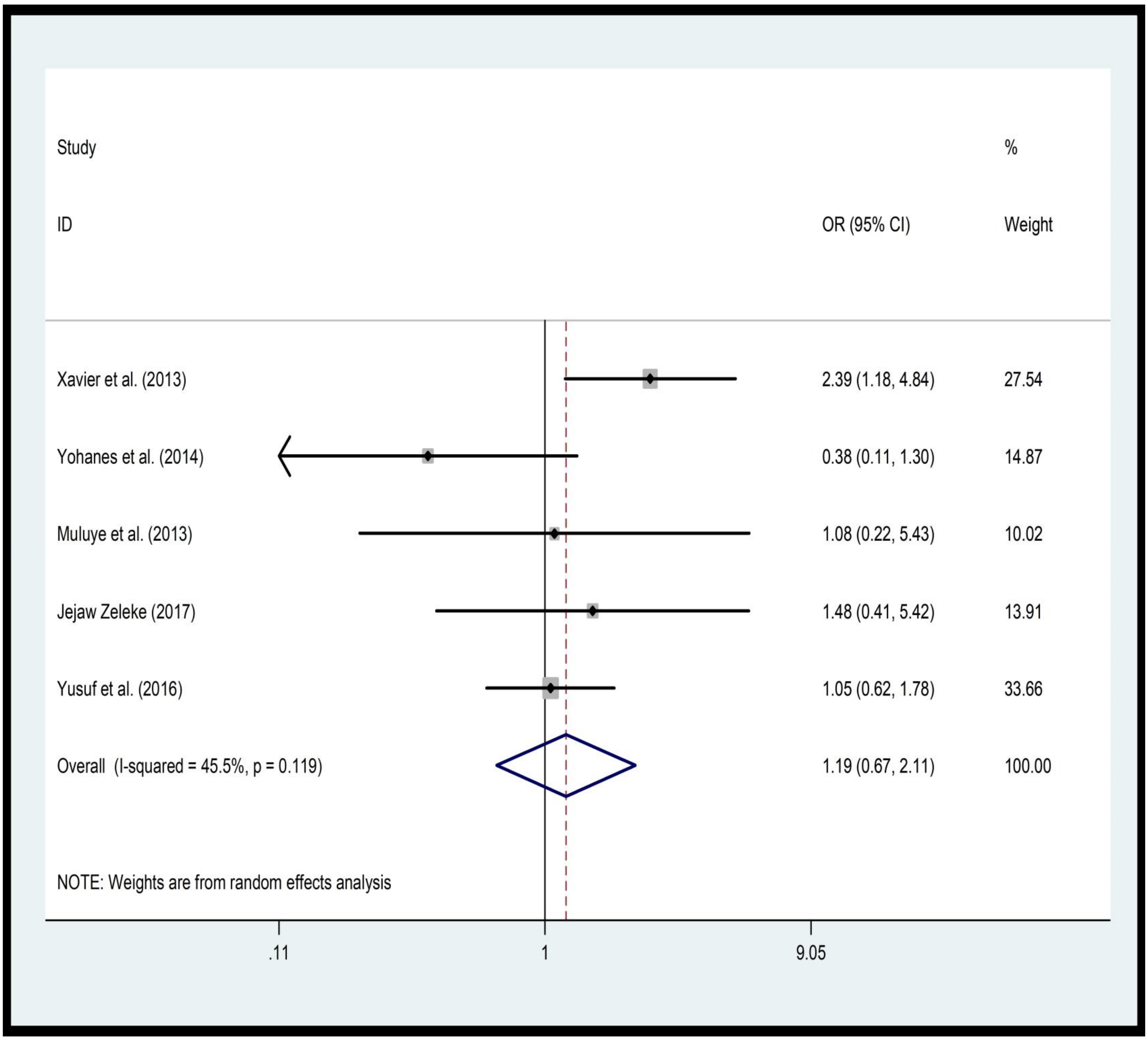

Figure S15. Forest plots of pooled odds ratio (OR) for latent *T. gondii* infection in HIV^+ people with respect to drinking untreated water. HIV^+ people who drank treated water were considered as the reference category to estimate OR.
