# Supplementary material for: Seroprevalence Estimates of Latent and Acute Toxoplasma Infections in HIV+ People—Call for Action in Underprivileged Communities
Source: Microorganisms. 2021 Sep 26;9(10):2034. doi: 10.3390/microorganisms9102034 (PMC8537859; doi:10.3390/microorganisms9102034)
Supplement: Supplementary file 1 [file microorganisms-09-02034-s001.zip › Supplementary Tables.pdf]

Table S1. Main features of studies included on the seroprevalences of latent and acute *Toxoplasma gondii* infection (LT and AT, respectively) in HIV+ people worldwide

Author/Year/WHO region	Reference	Study type	Country	Diagnostic method used for LT	Diagnostic method used for AT	Human Development Index (HDI)	Income level	Risk of bias	No. of HIV+ people screened (total)	No. of HIV+ people with LT	No. of HIV+ people with AT
**South America**											
Chaves-Borges et al. (1999)	[1]	CS	Brazil	ELISA	IgG & IgM	High	UM	Moderate	96	22	4
Lago et al. (2009)	[2]	CS	Brazil	ELFA	NA	High	UM	Low	168	103	na
Vidal et al. (2011)	[3]	CC	Brazil	IFAT	NA	High	UM	Low	192	130	na
Fernandes et al. (2012)	[4]	CC	Brazil	ELISA	Seroconversion	High	UM	Moderate	82	49	3
Xavier et al. (2013)	[5]	CS	Brazil	IFAT	IgG & IgM	High	UM	Low	250	200	0
Perez et al. (2009)	[6]	RC	Chile	ELISA	NA	Very high	High	Low	255	67	na
Maia et al. (2015)	[7]	CS	Brazil	ELISA	NA	High	UM	Low	658	10	na
da Silva et al. (2017)	[8]	CS	Brazil	ELISA	NA	High	UM	Low	769	447	na
da Silva et al. (2020)	[9]	CS	Brazil	ELISA	IgG & IgM	High	UM	Low	435	242	13
**African region**											
Quinn et al. (1987)	[10]	CC	DR Congo	IFAT	NA	Low	Low	Moderate	38	28	na
Carme et al. (1988)	[11]	CS	Congo	ELISA	NA	medium	Low	Low	375	75	na
Zumla et al. (1991)	[12]	CC	Uganda	SFT	NA	Low	Low	Low	186	64	na
Zumla et al. (1991)	[12]	CC	Zambia	SFT	NA	Medium	LM	Low	187	8	na
Ledru et al. (1995)	[13]	CS	Burkina Faso	ELISA	NA	Low	Low	Moderate	85	39	na
Woldemichael et al. (1998)	[14]	PC	Ethiopia	SFT	NA	Low	Low	Low	170	136	na
Millogo et al. (2000)	[15]	CS	Burkina Faso	ELISA	NA	Low	Low	Low	1828	464	na
Alonso et al. (2002)	[16]	RC	Canary island (Spain)	MEIA	IgG & IgM	Very high	High	Low	157	56	1
Uneke et al. (2005)	[17]	CC	Nigeria	ELISA	NA	Low	LM	Low	219	85	na
Lindstrom et al. (2006)	[18]	CS	Uganda	DAT	NA	Low	Low	Low	130	70	na
Simpore et al. (2006)	[19]	CS	Burkina Faso	ELISA	IgG & IgM	Low	Low	Low	207	59	0
Hari et al. (2007)	[20]	PC	South Africa	ELISA	IgG & IgM	Medium	UM	Low	307	24	3
Wester et al. (2006)	[21]	CS	Botswana	ELISA	IgG & IgM	High	UM	Moderate	46	3	0
Ouremi et al. (2009)	[22]	CS	Burkina Faso	ELISA	NA	Low	Low	Low	138	44	na
Shimelis et al. (2009)	[23]	CC	Ethiopia	ELISA	NA	Low	Low	Low	165	154	na
Sitoe et al. (2010)	[24]	CS	Mozambique	ELISA	NA	Low	Low	Moderate	58	18	na
Huruy et al. (2010)	[25]	RC	Ethiopia	ELISA	NA	Low	Low	Low	170	22	na
Oshinaike et al. (2010)	[26]	CC	Nigeria	ELISA	NA	Low	LM	Moderate	83	71	na
Assob et al. (2011)	[27]	CS	Cameroon	ELISA	IgG & IgM	Low	LM	Low	133	93	3
Osunkalu et al. (2011)	[28]	CS	Nigeria	ELISA	NA	Low	LM	Low	380	206	na
Pennap et al. (2011)	[29]	CS	Nigeria	ELISA	NA	Low	LM	Low	184	32	na
Goni et al. (2012)	[30]	CS	Nigeria	ELISA	NA	Low	LM	Low	110	21	na
Amuta et al. (2012)	[31]	CS	Nigeria	ELISA	NA	Low	LM	Low	360	39	na
Aleme et al. (2013)	[32]	CS	Ethiopia	ELISA	IgG & IgM	Low	Low	Low	150	141	0
Uttah et al. (2013)	[33]	CS	Nigeria	ELISA	NA	Low	LM	Moderate	72	20	na
Domingos et al. (2013)	[34]	CS	Mozambique	LAT	NA	Low	Low	Low	200	92	na
Muluye et al. (2013)	[35]	CS	Ethiopia	LAT	NA	Low	Low	Low	170	130	na
Ogoina et al. (2013)	[36]	CS	Nigeria	ELISA	IgG & IgM	Low	LM	Low	111	42	1
Walle et al. (2013)	[37]	CS	Ethiopia	ELISA	NA	Low	Low	Low	103	90	na
Endris et al. (2014)	[38]	CS	Ethiopia	ELISA	NA	Low	Low	Moderate	43	38	na
Okwuzu et al. (2014)	[39]	CS	Nigeria	ELISA	NA	Low	LM	Low	242	100	na
Yohanes et al. (2014)	[40]	CS	Ethiopia	ELISA	NA	Low	Low	Low	170	150	na
Yesuf and Melese, (2015)	[41]	CS	Ethiopia	ELISA	NA	Low	Low	Low	120	72	na
Wam et al. (2016)	[42]	CS	Cameroon	ELISA	IgG & IgM	Low	LM	Low	90	43	11
Yusuf et al. (2016)	[43]	CS	Nigeria	ELISA	NA	Low	Low	Low	273	84	na
Tegegne et al. (2016)	[44]	CS	Ethiopia	LAT	NA	Low	Low	Low	135	109	na
Sanyaolu et al. (2016)	[45]	CS	Nigeria	ELISA	NA	Low	LM	Moderate	65	2	na
Mirambo et al. (2016)	[46]	CS	Tanzania	ELISA	NA	Low	Low	Moderate	38	26	na
Ayi et al. (2016)	[47]	CS	Ghana	ELISA	IgG & IgM	Medium	LM	Low	125	72	0
Frimpong et al. (2017)	[48]	CS	Zambia	ELISA	IgG & IgM	Medium	LM	Moderate	69	6	0
Pappoe et al. (2017)	[49]	CS	Ghana	ELISA	IgG & IgM	Medium	LM	Low	394	293	1

Ngobeni and Samie (2017)	[50]	CS	South Africa	ELISA	IgG & IgM	Medium	UM	Low	100	38	4
Bamba et al. (2017)	[51]	CS	Burkina Faso	ELISA	IgG & IgM	Low	Low	Low	290	85	0
Jejaw Zeleke et al. (2017)	[52]	CS	Ethiopia	ELISA	IgG & IgM	Low	Low	Low	270	255	6
Katawa et al. (2018)	[53]	CS	Togo	ELISA	IgG & IgM	Low	Low	Moderate	56	14	2
Olaniyan et al. (2019)	[54]	CC	Nigeria	ELISA	NA	Low	LM	Moderate	50	7	na
Abongwa et al. (2019)	[55]	CS	Cameroon	ELISA	NA	Low	LM	Moderate	70	31	na
**Eastern Mediterranean region**											
Yousif et al. (1994)	[56]	CS	Bahrain	ELISA	IgG & IgM	Very high	High	Moderate	76	16	0
Davarpanah et al. (2007)	[57]	CS	Iran	ELISA	NA	High	UM	Low	208	38	na
Shafiei et al. (2011)	[58]	CS	Iran	ELISA	IgG & IgM	High	UM	Low	121	46	3
Daryani et al. (2011)	[59]	CS	Iran	ELISA	IgG & IgM	High	UM	Moderate	62	48	6
Mohraz et al. (2011)	[60]	CS	Iran	ELISA	NA	High	UM	Low	201	100	na
Addebbous et al. (2012)	[61]	CS	Morocco	ELISA	NA	Medium	LM	Low	95	59	na
Abdollahi et al. (2013)	[62]	CC	Iran	ELISA	NA	High	UM	Low	100	65	na
Khalil et al. (2013)	[63]	CS	Sudan	LAT	NA	Low	LM	Moderate	44	33	na
Alavi et al. (2013)	[64]	CC	Iran	ELISA	NA	High	UM	Moderate	42	31	na
Rostami et al. (2014)	[65]	CS	Iran	ELISA	IgG & IgM	High	UM	Low	94	18	1
Rahimi et al. (2015)	[66]	CS	Iran	ELISA	IgG & IgM	High	UM	Moderate	82	79	0
Zaini et al. (2016)	[67]	CC	Saudi Arabia	ELISA	IgG & IgM	Very high	High	Moderate	50	15	9
Rezanezhad et al. (2017)	[68]	CS	Iran	ELISA	NA	High	UM	Low	90	19	na
Aghaee et al. (2017)	[69]	CS	Iran	ELISA	IgG & IgM	High	UM	Moderate	49	10	1
Aghakhani et al. (2018)	[70]	CS	Iran	ELISA	IgG & IgM	High	UM	Low	93	42	2
Shafieenia et al. (2018)	[71]	CS	Iran	ELISA	IgG & IgM	High	UM	Low	379	131	12
Ebrahim Saraie et al. (2018)	[72]	CS	Iran	ELISA	NA	High	UM	Low	246	51	na
Arefkhah et al. (2018)	[73]	CS	Iran	ELISA	IgG & IgM	High	UM	Low	251	39	0
Nazari et al. (2018)	[74]	CS	Iran	ELISA	IgG & IgM	High	UM	Low	385	157	10
Bavand et al. (2019)	[75]	CS	Iran	ELISA	IgG & IgM	High	UM	Low	149	69	4
Ahmadpour et al. (2019)	[76]	CS	Iran	ELISA	IgG & IgM	High	UM	Low	124	47	2
Bokharaei-Salim et al. (2019)	[77]	CS	Iran	ELISA	IgG & IgM	High	UM	Low	108	88	1
Hosseini et al. (2020)	[78]	CS	Iran	ELISA	IgG & IgM	High	UM	Low	102	70	0
**European region**											
Holliman (1990)	[79]	CS	UK	SFT	Seroconversion	Very high	High	Low	500	133	7
Aspock et al. (1990)	[80]	PC	Austria	IFAT	NA	Very high	High	Low	618	358	na
Zangerle et al. (1991)	[81]	PC	Austria	IFAT	NA	Very high	High	Moderate	41	19	na
Sykora et al. (1992)	[82]	CC	Czech Republic	CFT	IgG & IgM	Very high	High	Moderate	67	20	0
Zufferey et al. (1993)	[83]	PC	Switzerland	IFAT	Seroconversion	Very high	High	Low	715	360	12
Huengsberg et al. (1995)	[84]	CC	UK	LAT	NA	Very high	High	Low	109	31	na
Champs et al. (1997)	[85]	CS	France	ELISA	IgG & IgM	Very high	High	Moderate	32	22	0
Djurkovic-Djakovic et al. (1997)	[86]	RC	Serbia	ELISA	IgG & IgM	Very high	High	Low	288	127	0
Garly et al. (1997)	[87]	CS	Denmark	IFAT	Seroconversion	Very high	High	Low	503	223	4
Reiter- Owona et al. (1998)	[88]	RC	Germany	SFT	Seroconversion	Very high	High	Low	183	64	6
Boto de los Bueis et al. (1998)	[89]	CC	Spain	MEIA	NA	Very high	High	Low	255	93	na
Belanger et al. (1999)	[90]	RC	France	ELISA	Seroconversion	Very high	High	Low	1683	1215	14
San-Andre´s et al. (2003)	[91]	RC	Spain	ELISA	NA	Very high	High	Low	1018	422	na
Llenas-García et al. (2012)	[92]	RC	Spain	ELISA	NA	Very high	High	Low	371	13	na
Csep and Drăghici (2013)	[93]	CS	Romania	ELISA	Seroconversion	Very high	High	Moderate	30	6	2
Kodym et al. (2015)	[94]	RC	Czech Republic	ELISA	Seroconversion	Very high	High	Low	1235	502	14
Dakovic Rode et al. (2010)	[95]	CC	Croatia	ELISA	IgG & IgM	Very high	High	Low	166	86	2
Aydin et al. (2011)	[96]	CS	Turkey	ELISA	IgG & IgM	High	UM	Low	164	85	0
Ene et al. (2016)	[97]	CS	Romania	ELISA	IgG & IgM	Very high	High	Low	194	63	0
Senoglu et al. (2018)	[98]	RC	Turkey	ELISA	IgG & IgM	High	UM	Low	614	267	0
**North and Caribbean America**											

Quinn et al. (1987)	[10]	CC	USA	IFAT	IgG & IgM	Very high	High	Moderate	60	38	6
Israelski et al. (1993)	[99]	CS	USA	DAT	Seroconversion	Very high	High	Low	443	42	4
Grant et al. (1990)	[100]	CS	USA	IFAT	Seroconversion	Very high	High	Low	411	130	4
Wallace etal. (1993)	[101]	RC	USA	ELISA	Seroconversion	Very high	High	Low	723	70	13
Fachado et al. (1994)	[102]	PC	Cuba	ELISA	NA	High	UM	Moderate	79	56	na
Mathews (1994)	[103]	CS	USA	ELISA	NA	Very high	UM	Low	344	55	na
Galvan Ramirez et al. (1997)	[104]	CS	Mexico	ELISA	IgG & IgM	High	UM	Low	92	46	1
Gongora-Biachi et al. (1998)	[105]	CC	Mexico	MEIA	NA	High	UM	Low	95	45	na
Johns and Gill (1998)	[106]	PC	Canada	MEIA	NA	Very high	High	Low	1074	114	na
Falusi et al. (2002)	[107]	CS	USA	SFT	NA	Very high	High	Low	1973	301	na
Bharti et al. (2016)	[108]	CS	USA	ELISA	NA	Very high	High	Low	263	30	na
O'Bryan et al. (2016)	[109]	RC	USA	ELISA	NA	Very high	High	Low	1645	223	na
**South-East Asia region**					NA						na
Meisheri et al. (1997)	[110]	CC	India	ELISA	NA	Medium	LM	Moderate	89	60	na
Sukthana et al. (2000)	[111]	CC	Thailand	ELISA	NA	High	UM	Low	190	44	na
Wanachiwanawin et al. (2001)	[112]	CC	Thailand	ELISA	IgG & IgM	High	UM	Low	838	450	3
Praharaj et al. (2001)	[113]	CC	India	ELISA	NA	Medium	LM	Moderate	80	18	na
Malla et al. (2005)	[114]	CC	India	ELISA	IgG & IgM	Medium	LM	Low	100	12	2
Sucilathangam et al. (2012)	[115]	CC	India	ELISA	NA	Medium	LM	Low	160	24	na
Bhattacharyya et al. (2013)	[116]	CS	India	ELISA	NA	Medium	LM	Low	100	42	na
Anuradha and Preethi (2014)	[117]	CS	India	ELISA	NA	Medium	LM	Low	92	32	na
Chemoh et al (2015)	[118]	CS	Thailand	ELISA	IgG avidity	High	UM	Low	300	109	26
Haryati et al. (2015)	[119]	CS	Indonesia	ELISA	NA	Medium	LM	Low	306	97	na
Prasetyo et al. (2015)	[120]	RC	Indonesia	ELISA	IgG & IgM	Medium	LM	Low	597	260	23
Sari et al. (2015)	[121]	CC	Indonesia	ELISA	IgG & IgM	Medium	LM	Low	140	56	6
Uppal et al. (2015)	[122]	CS	India	ELISA	IgG avidity	Medium	LM	Low	661	141	2
Singh et al. (2015)	[123]	RC	India	ELISA	NA	Medium	LM	Low	729	81	na
Kasthuri (2018)	[124]	PC	India	ELISA	Seroconversion	Medium	LM	Low	207		5
Pimpalkar et al. (2019)	[125]	PC	India	ELISA	IgG & IgM	Medium	LM	Low	362	71	14
Garg et al. (2019)	[126]	CS	India	ELISA	IgG & IgM	Medium	LM	Low	400	51	4
Halleyantoro et al. (2019)	[127]	CS	Indonesia	ELISA	NA	Medium	LM	Moderate	88	34	na
**Western Pacific region**											
Yoong et al. (1997)	[128]	CS	Malaysia	ELISA	IgG & IgM	Very high	UM	Moderate	49	24	1
Oh et al. (1999)	[129]	CS	South korea	ELISA	NA	Very high	High	Low	173	7	na
Hagiwara et al. (2001)	[130]	RC	Japan	ELISA	NA	Very high	High	Moderate	56	7	na
Zhou et al. (2001)	[131]	CC	China	MEIA	NA	High	UM	Moderate	50	13	na
Nissapatorn et al. (2002)	[132]	CS	Malaysia	ELISA	NA	Very high	UM	Low	100	21	na
Nissapatorn et al. (2003)	[133]	RC	Malaysia	ELISA	NA	Very high	UM	Low	419	32	na
Nissapatorn et al. (2003)	[133]	CS	Malaysia	ELISA	NA	Very high	UM	Low	301	124	na
Nissapatorn et al. (2004)	[134]	RC	Malaysia	ELISA	NA	Very high	UM	Low	505	226	na
Hung et al. (2005)	[135]	CS	Taiwan	ELISA	NA	very high	High	Low	550	56	na
Naito et al. (2007)	[136]	CS	Japan	ELISA	NA	Very high	High	Moderate	56	3	na
Zhang et al. (2008)	[137]	PC	China	ELISA	NA	High	UM	Low	168	13	na
Hua et al. (2009)	[138]	CC	China	ELISA	NA	High	UM	Low	259	25	na
Tian et al. (2010)	[139]	CC	China	ELISA	NA	High	UM	Low	309	3	na
Song (2012)	[140]	CS	China	ELISA	NA	High	UM	Moderate	50	5	na
You et al. (2012)	[141]	CS	China	ELISA	NA	High	UM	Low	927	325	na
John et al. (2012)	[142]	CC	Papua New Guinea	ELISA	NA	Low	LM	Low	181	108	na
Lim et al. (2013)	[143]	CS	Singapore	ELISA	NA	Very high	High	Low	771	183	na
Takahashi et al. (2014)	[144]	RC	Japan	ELISA	NA	Very high	High	Low	169	24	na
Pang et al. (2015)	[145]	CS	China	ELISA	NA	High	UM	Low	450	32	na
Angal et al. (2016)	[146]	CC	Malaysia	ELISA	IgG & IgM	Very high	UM	Low	133	84	2
Chen et al. (2016)	[147]	RC	China	ELISA	NA	High	UM	Low	342	84	na
Shen et al. (2016)	[148]	CC	China	ELISA	IgG & IgM	High	UM	Low	259	25	3
Pang et al. (2018)	[149]	CS	China	ELISA	NA	High	UM	Low	954	73	na
Hoshina et al. (2019)	[150]	CS	Japan	ELISA	NA	Very high	High	Low	399	33	na

Abbreviation: na, not assessed; **CC**, case-control; **CS**, cross sectional; **RC**, retrospective cohort; **PC**, prospective cohort; **LM**, lower middle; **UM**, upper middle; **ELISA**, enzyme-linked immunosorbent assay; **IFA**, immunofluorescence assay; **MAT**, modified agglutination test; **ELFA**, enzyme-linked fluorescent assay technique; **LAT**, latex agglutination test; **SFT**, Sabin-Feldman test; **IMx**

immunoassay, Abbott Toxo immunoglobulin G (IgG) assay; **MEIA**, microparticle enzyme immunoassay; **LFIA**, Lateral flow immunoassay assay; **DAT**, direct agglutination test; **DR** Congo, Democratic Republic of the Congo.

References

1. Chaves-Borges, F.A.; Souza, M.A.; Silva, D.A.; Kasper, L.H.; Mineo, J.R. Detection of *Toxoplasma gondii* soluble antigen, SAG-1(p30), antibody and immune complex in the cerebrospinal fluid of HIV positive or negative individuals. *Rev. Inst. Med. Trop. Sao Paulo* **1999**, *41*, 329–338.
2. Lago, E.; Conrado, G.; Piccoli, C.; Carvalho, R.; Bender, A. *Toxoplasma gondii* antibody profile in HIV-infected pregnant women and the risk of congenital toxoplasmosis. *Eur. J. Clin. Microbiol. Infect. Dis.* **2009**, *28*, 345–351.
3. Vidal, J.E.; Diaz, A.V.; de Oliveira, A.C.; Dauar, R.F.; Colombo, F.A.; Pereira-Chioccola, V.L. Importance of high IgG anti-*Toxoplasma gondii* titers and PCR detection of T. gondii DNA in peripheral blood samples for the diagnosis of AIDS-related cerebral toxoplasmosis: a case-control study. *Braz. J. Infect. Dis.* **2011**, *15*, 356–359.
4. Fernandes, M.A.; Batista, G.I.; Carlos, J.d.C.S.; Gomes, I.M.; Azevedo, K.M.L.d.; Setúbal, S.; Oliveira, S.A.d.; Velarde, L.G.C.; Cardoso, C.A.A. *Toxoplasma gondii* antibody profile in HIV-1-infected and uninfected pregnant women and the impact on congenital toxoplasmosis diagnosis in Rio de Janeiro, Brazil. *Braz. J. Infect. Dis.* **2012**, *16*, 170–174.
5. Xavier, G.A.; Cademartori, B.G.; Cunha Filho, N.A.d.; Farias, N.A.d.R. Evaluation of seroepidemiological toxoplasmosis in HIV/AIDS patients in the south of Brazil. *Rev. Inst. Med. Trop. Sao Paulo* **2013**, *55*, 25–30.
6. Pérez, C.; Cerón, A.; Fuentes, L.; Zañartu, S.; Balcells, M.; Ajenjo, H.; Rabagliati, B.; Labarca, L.; Acuña, L. Hepatitis B, C, Treponema pallidum and *Toxoplasma gondii* co-infections in HIV infected patients. *Rev. Med. Chil.* **2009**, *137*, 8.
7. Maia, M.; Lage, E.M.; Moreira, B.; Deus, E.; Faria, J.G.; Pinto, J.A.; Melo, V.H. Prevalence of congenital and perinatal infection in HIV positive pregnant in Belo Horizonte metropolitan region. *Rev. Bras. Ginicol. Obstet.* **2015**, *37*, 421–427.
8. da Silva, C.M.; de Peder, L.D.; Menolli, R.A.; Takizawa, M.d.G.M.H.; Takizawa, M.C.H.; Horvath, J.D.; Silva, E.S.; Teixeira, J.J.V.; Bertolini, D.A. *Toxoplasma gondii*–soroprevalência em patients HIV no sul do Brasil. *Saúde (Santa Maria).* **2017**, *43*, 73–80.
9. da Silva, B.E.B.; Santos, V.S.; Santos, I.E.R.; Batista, M.V.d.A.; Gonçalves, L.L.C.; Lemos, L.M.D.d. Prevalence of coinfections in women living with human immunodeficiency virus in Northeast Brazil. *Rev. Soc. Bras. Med.* **2020**, *53*, e20190282.
10. Quinn, T.C.; Piot, P.; McCormick, J.B.; Feinsod, F.M.; Taelman, H.; Kapita, B.; Stevens, W.; Fauci, A.S. Serologic and immunologic studies in patients with AIDS in North America and Africa: the potential role of infectious agents as cofactors in human immunodeficiency virus infection. *JAMA* **1987**, *257*, 2617–2621.
11. Carme, B.; M'Pele, P.; Mbitsi, A.; Kissila, A.; Aya, G.; Mouanga-Yidika, G.; Mboussa, J.; Itoua-Ngaporo, A. Opportunistic parasitic diseases and mycoses in AIDS. Their frequencies in Brazzaville (Congo). *Bull. Soc. Pathol. Exot. Filiales* **1988**, *81*, 311–316.
12. Zumla, A.; Savva, D.; Wheeler, R.B.; Hira, S.; Luo, N.; Kaleebu, P.; Sempala, S.; Johnson, J.D.; Holliman, R. *Toxoplasma* serology in Zambian and Ugandan patients infected with the human immunodeficiency virus. *Trans. R. Soc. Trop. Med. Hyg.* **1991**, *85*, 227–229.
13. Ledru, E.; Diagbouga, S.; Ledru, S.; Cauchoix, B.; Yameogo, M.; Chami, D.; Sanou, O.; Chiron, J.P. A study of *Toxoplasma* and Cytomegalovirus serology in tuberculosis and in HIV-infected patients in Burkina Faso. *Acta Trop.* **1995**, *59*, 149–154.
14. Woldemichael, T.; Fontanet, A.L.; Sahlu, T.; Gilis, H.; Messele, T.; Rinke de Wit, T.F.; Yeneneh, H.; Coutinho, R.A.; Van Gool, T. Evaluation of the Eiken latex agglutination test for anti-*Toxoplasma* antibodies and seroprevalence of *Toxoplasma* infection among factory workers in Addis Ababa, Ethiopia. *Trans. R. Soc. Trop. Med. Hyg.* **1998**, *92*, 401–403.
15. Millogo, A.; Ki-Zerbo, G.A.; Traore, W.; Sawadogo, A.B.; Ouedraogo, I.; Peghini, M. [*Toxoplasma* serology in HIV infected patients and suspected cerebral toxoplasmosis at the Central Hospital of Bobo-Dioulasso (Burkina Faso)]. *Bull. Soc. Pathol. Exot.* **2000**, *93*, 17–19.
16. Alonso, R.; Martinez, E.; Laynez, P.; Miguelez, M.; Pinero, J.E.; Valladares, B. Detection by nested-PCR of *Toxoplasma gondii* in patients infected with human immunodeficiency virus. *Med. Clin (Barc).* **2002**, *118*, 294–296.
17. Uneke, C.J.; Duhlinska, D.D.; Njoku, M.O.; Ngwu, B.A. Seroprevalence of acquired toxoplasmosis in HIV-infected and apparently healthy individuals in Jos, Nigeria. *Parassitologia* **2005**, *47*, 233–236.
18. Lindstrom, I.; Kaddu-Mulindwa, D.H.; Kironde, F.; Lindh, J. Prevalence of latent and reactivated *Toxoplasma gondii* parasites in HIV-patients from Uganda. *Acta Trop.* **2006**, *100*, 218–222.
19. Simpore, J.; Savadogo, A.; Ilboudo, D.; Nadambega, M.C.; Esposito, M.; Yara, J.; Pignatelli, S.; Pietra, V.; Musumeci, S. *Toxoplasma gondii*, HCV, and HBV seroprevalence and co-infection among HIV-positive and-negative pregnant women in Burkina Faso. *J. Med. Virol.* **2006**, *78*, 730–733.
20. Hari, K.R.; Modi, M.R.; Mochan, A.H.; Modi, G. Reduced risk of *Toxoplasma* encephalitis in HIV-infected patients--a prospective study from Gauteng, South Africa. *Int. J. STD AIDS* **2007**, *18*, 555–558.
21. Wester, C.W.; Bussmann, H.; Moyo, S.; Avalos, A.; Gaolathe, T.; Ndwapi, N.; Essex, M.; MacGregor, R.R.; Marlink, R.G. Serological evidence of HIV-associated infection among HIV-1—infected adults in Botswana. *Clin. Infect. Dis.* **2006**, *43*, 1612–1615.
22. Ouermi, D.; Simpore, J.; Belem, A.; Sanou, D.; Karou, D.; Ilboudo, D.; Bisseye, C.; Onadja, S.; Pietra, V.; Pignatelli, S. Co-infection of *Toxoplasma gondii* with HBV in HIV-infected and uninfected pregnant women in Burkina Faso. *Pak. J. Biol. Sci.* **2009**, *12*, 1188.
23. Shimelis, T.; Tebeje, M.; Tadesse, E.; Tegbaru, B.; Terefe, A. Sero-prevalence of latent *Toxoplasma gondii* infection among HIV-infected and HIV-uninfected people in Addis Ababa, Ethiopia: A comparative cross-sectional study. *BMC Res. Notes.* **2009**, *2*, 213.
24. Sitoe, S.P.B.L.; Rafael, B.; Meireles, L.R.; Andrade Jr, H.F.d.; Thompson, R. Preliminary report of HIV and *Toxoplasma gondii* occurrence in pregnant women from Mozambique. *Rev. Inst. Med. Trop. São Paulo* **2010**, *52*, 291–295.
25. Huruy, K.; Kassu, A.; Mulu, A.; Wondie, Y. Immune restoration disease and changes in CD4+ T-cell count in HIV- infected patients during highly active antiretroviral therapy at Zewditu memorial hospital, Addis Ababa, Ethiopia. *AIDS Res. Ther.* **2010**, *7*, 46.
26. Oshinaike, O.O.; Okubadejo, N.U.; Ojini, F.I.; Danesi, M.A. A preliminary study of the frequency of focal neurological deficits in HIV/AIDS patients seropositive for *Toxoplasma gondii* IgG in Lagos, Nigeria. *Nig. Q. J. Hosp. Med.* **2010**, *20*, 104–107.
27. Assob, J.C.; Njunda, A.L.; Nsagha, D.S.; Kamga, H.L.; Weledji, P.E.; Che, V. *Toxoplasma* antibodies amongst HIV/AIDS patients attending the University Teaching Hospital Yaounde in Cameroon. *Afr. J. Clin. Exp. Microbiol.* **2011**, *12*, 1119–1123.
28. Osunkalu, V.O.; Akanmu, S.A.; Ofomah, N.J.; Onyiaorah, I.V.; Adediran, A.A.; Akinde, R.O.; Onwuezobe, I.A. Seroprevalence of *Toxoplasma gondii* IgG antibody in HIV-infected patients at the Lagos University Teaching Hospital. *HIV AIDS (Auckl)* **2011**, *3*, 101–105.
29. Pennap, G.R.; Taiye, E.; Adoga, M. Seroprevalence of *Toxoplasma gondii* infection among people living with HIV/AIDS in Keffi. *J. Antivir. Antiretrovir.* **2011**, *3*, 38.
30. Goni, B.; Bakki, B.; Kida, I.; Yusuph, H.; Zailani, S.; Mustapha, S.; Tahir, A.; Sahabi, M.; Talle, M.; Baba, M. Seroprevalence of IgG anti-T. gondii antibody among HIV infected patients in Maiduguri, North eastern Nigeria. *J. Med. Sci.* **2012**, *6*, 7–14.
31. Amuta, E.; Amali, O.; Jacob, S.; Houmsou, R. *Toxoplasma gondii* IgG antibodies in HIV/AIDS patients attending hospitals in Makurdi metropolis, Benue state, Nigeria. *Int. J. Med. Biomed. Res.* **2012**, *1*, 186–192.

32. Aleme, H.; Tilahun, G.; Berhe, D.F.N.; Medhin, G. Seroprevalence of immunoglobulin-G and of immunoglobulin-M anti-*Toxoplasma gondii* antibodies in human immunodeficiency virus infection/acquired immunodeficiency syndrome patients at Tikur Anbessa Specialized Hospital, Addis Ababa, Ethiopia. *J. Infect. Dis. Ther.* **2013**, *1*, 119.
33. Uttah, E.C.; Ajang, R.; Ogbeche, J.; Etta, H.; Etim, L. Comparative seroprevalence and risk factors of toxoplasmosis among three subgroups in Nigeria. *J. Nat. Sci. Res.* **2013**, *3*, 23–28.
34. Domingos, A.; Ito, L.S.; Coelho, E.; Lucio, J.M.; Matida, L.H.; Ramos, A.N., Jr. Seroprevalence of *Toxoplasma gondii* IgG antibody in HIV/AIDS-infected individuals in Maputo, Mozambique. *Rev. Saude Publica* **2013**, *47*, 890–896.
35. Muluye, D.; Wondimeneh, Y.; Belyhun, Y.; Moges, F.; Endris, M.; Ferede, G.; Yitayew, G.; Negese, D. Prevalence of *Toxoplasma gondii* and Associated Risk Factors among People Living with HIV at Gondar University Hospital, Northwest Ethiopia. *ISRN Trop. Med.* **2013**, *2013*, 5.
36. Ogoina, D.; Onyemelukwe, G.C.; Musa, B.O.; Obiako, R.O. Seroprevalence of IgM and IgG antibodies to *Toxoplasma* infection in healthy and HIV-positive adults from Northern Nigeria. *J. Infect. Dev. Ctries.* **2013**, *7*, 398–403.
37. Walle, F.; Kebede, N.; Tsegaye, A.; Kassa, T. Seroprevalence and risk factors for Toxoplasmosis in HIV infected and non-infected individuals in Bahir Dar, Northwest Ethiopia. *Parasites Vector* **2013**, *6*, 15.
38. Endris, M.; Belyhun, Y.; Moges, F.; Adefiris, M.; Tekeste, Z.; Mulu, A.; Kassu, A. Seroprevalence and associated risk factors of *Toxoplasma gondii* in pregnant women attending in Northwest Ethiopia. *Iran J. Parasitol.* **2014**, *9*, 407–414.
39. Okwuzu, J.; Odunukwe, N.; Ezechi, O.; Gbajabiamila, T.; Musa, A.; Ezeobi, P.; Oke, B.; Somefun, T.; Okoye, R.; Onyeitu, C. *Toxoplasma gondii* infection in HIV/AIDS: prevalence and risk factors. *Afr. J. Clin. Experiment. Microbiol.* **2014**, *15*, 97–102.
40. Yohanes, T.; Debalke, S.; Zemene, E. Latent *Toxoplasma gondii* Infection and Associated Risk Factors among HIV-Infected Individuals at Arba Minch Hospital, South Ethiopia. *AIDS Res. Treat.* **2014**, *2014*, 652941.
41. Yesuf, K.M.; Melese, Z.T. Prevalence of Toxoplasmosis in HIV/AIDS patients in Mettu Karl hospital. *Am. J. Heal Res.* **2015**, *3*, 183–188.
42. Wam, E.C.; Sama, L.F.; Ali, I.M.; Ebile, W.A.; Aghangu, L.A.; Tume, C.B. Seroprevalence of *Toxoplasma gondii* IgG and IgM antibodies and associated risk factors in women of child-bearing age in Njinikom, NW Cameroon. *BMC Res. Notes* **2016**, *9*, 406.
43. Yusuf, A.; Yahaya, S.; Azeez-Akande, O. Seroprevalence and risk factors of *Toxoplasma gondii* infection (toxoplasmosis) among HIV seropositive pregnant women in a tertiary healthcare centre, Kano, Northern Nigeria. *J. Med. Med. Sci.* **2016**, *7*, 1–5.
44. Tegegne, D.; Abdurahaman, M.; Mosissa, T.; Yohannes, M. Anti-*Toxoplasma* antibodies prevalence and associated risk factors among HIV patients. *Asian Pac. J. Trop. Med.* **2016**, *9*, 460–464.
45. Sanyaolu, A.; Oyibo, W.; Iriemenam, N.; Badaru, O. Sero-prevalence study of parasitic infections among HIV positive and negative patients in Lagos, Nigeria. *Afr. J. Clin. Experiment. Microbiol.* **2016**, *17*, 88–96.
46. Mirambo, M.M.; Kivambe, C.; Mushi, M.F.; Zinga, M.; Mngumi, E.B.; Mtebe, M.; Mshana, S.E. High seroprevalence of specific *Toxoplasma gondii* IgG antibodies among HIV/AIDS patients with immunological failure attending a tertiary hospital in northwestern Tanzania. *Tanzania J. Health Res.* **2016**, *18*.
47. Ayi, I.; Sowah, A.O.-K.; Blay, E.A.; Suzuki, T.; Ohta, N.; Ayeh-Kumi, P.F. *Toxoplasma gondii* infections among pregnant women, children and HIV-seropositive persons in Accra, Ghana. *Trop. Med. Health* **2016**, *44*, 17.
48. Frimpong, C.; Makasa, M.; Sitali, L.; Michelo, C. Seroprevalence and determinants of toxoplasmosis in pregnant women attending antenatal clinic at the university teaching hospital, Lusaka, Zambia. *BMC Infect. Dis.* **2017**, *17*, 10.
49. Pappoe, F.; Cheng, W.; Wang, L.; Li, Y.; Obiri-Yeboah, D.; Nuvor, S.V.; Ambachew, H.; Hu, X.; Luo, Q.; Chu, D., et al. Prevalence of *Toxoplasma gondii* infection in HIV-infected patients and food animals and direct genotyping of *T. gondii* isolates, Southern Ghana. *Parasitol. Res.* **2017**, *116*, 1675–1685.
50. Ngobeni, R.; Samie, A. Prevalence of *Toxoplasma gondii* IgG and IgM and associated risk factors among hiv-positive and hiv-negative patients in vhembe district of South Africa. *Afr. J. Infect. Dis.* **2017**, *11*, 1–9.
51. Bamba, S.; Zoungrana, J.; Nikiema, Z.; Sondo, A.K.; Ndiaye, J.L.; Bretagne, S. Impact of alternative treatment approach for cerebral toxoplasmosis among HIV/AIDS patients from a resource-poor setting in Burkina Faso. *Ann. Parasitol.* **2017**, *63*, 173–181.
52. Zeleke, A.J.; Melsew, Y.A. Seroprevalence of *Toxoplasma gondii* and associated risk factors among HIV-infected women within reproductive age group at Mizan Aman General Hospital, Southwest Ethiopia: a cross sectional study. *BMC Res. Notes* **2017**, *10*, 70.
53. Katawa, G.; Kolou, M.; Nadjir, L.K.; Ataba, E.; Bomboma, G.; Karou, S.D. CD4 T-Lymphocytes Count in HIV-*Toxoplasma gondii* Co-Infected Pregnant Women Undergoing a Prevention of Mother-to-Child Transmission Program. *J. Biosci. Med.* **2018**, *6*, 76.
54. Olaniyan, M.F.; Kilo, O.A. Immunochemical evidence of Trypanosoma cruzi and *Toxoplasma gondii* parasitic infections in human immunodeficiency virus/acquired immunodeficiency syndrome subjects in relationship with the CD4+ count. *Imam J. Appl. Sci.* **2019**, *4*, 16.
55. Abongwa, L.E.; Signang, A.; Tibi, S.; Ngenwi, A. Socio-demographic and Obstetric Variations of *T. gondii* and HIV-1 Co-infection among Pregnant Women in Cameroon. *J. Adv. Microbiol.* **2019**, *16*, 1–11.
56. Yousif, A.; Wallace, M.; Baig, B. The seroprevalence of syphilis, toxoplasmosis and hepatitis B in patients in Bahrain infected with human immunodeficiency virus. *Trans. R. Soc. Trop. Med. Hyg.* **1994**, *88*, 60.
57. Davarpanah, M.; Mehrabani, D.; Neirami, R.; Ghahremanpoori, M.; Darvishi, M. Toxoplasmosis in HIV/AIDS patients in Shiraz,southern Iran. *Iran Red. Crescent Med. J.* **2007**, *9*, 22–27.
58. Shafiei, R.; Riazi, Z.; Sarvghad, M.; Galian Sharifdini, M.; Mahmoodzadeh, A.; Hajia, M. Prevalence of IgG and IgM anti-*Toxoplasma gondii* antibodies in HIV positive patients in northeast of Iran. *Iran J. Pathol.* **2011**, *6*, 68–72.
59. Daryani, A.; Sharif, M.; Meigouni, M. Seroprevalence of IgG and IgM anti-*Toxoplasma* antibodies in HIV/AIDS patients, northern Iran. *Asian Pac. J. Trop. Med.* **2011**, *4*, 271–274.
60. Mohraz, M.; Mehrkhani, F.; Jam, S.; SeyedAlinaghi, S.; Sabzvari, D.; Fattahi, F.; Jabbari, H.; Hajiabdolbaghi, M. Seroprevalence of toxoplasmosis in HIV(+)/AIDS patients in Iran. *Acta Med. Iran.* **2011**, *49*, 213–218.
61. Addebbous, A.; Adarmouch, L.; Tali, A.; Laboudi, M.; Amine, M.; Aajly, L.; Rhajaoui, M.; Chabaa, L.; Zougaghi, L. IgG anti-*Toxoplasma* antibodies among asymptomatic HIV-infected patients in Marrakesh-Morocco. *Acta Trop.* **2012**, *123*, 49–52.
62. Abdollahi, A.; Shoar, S.; Sheikhbahaei, S.; Jafari, S. Sero-prevalence of Cytomegalovirus and *Toxoplasma* infectionsamong newly diagnosed HIV patients in Iran; assessing the correlation with CD4+ cell counts. *Iran J. Pathol.* **2013**, *8*, 81–88.
63. Khalil, M.; Ahmed, A.A.; Elrayah, I.E. Prevalence and Risk factors for *Toxoplasma gondii* infection in Humans from Khartoum State, Sudan. *Int. J. Public Health Epidemiol.* **2013**, *2*, 60–66.
64. Alavi, S.M.; Jamshidian, R.; Salmanzadeh, S. Comparative study on *Toxoplasma* serology among HIV positive and HIV negative illicit drug users in Ahvaz, Iran. *Caspian J. Intern. Med.* **2013**, *4*, 781–784.
65. Rostami, A.; Keshavarz, H.; Shojaee, S.; Mohebali, M.; Meamar, A.R. Frequency of *Toxoplasma gondii* in HIV Positive Patients from West of Iran by ELISA and PCR. *Iran J. Parasitol.* **2014**, *9*, 474–481.
66. Rahimi, M.T.; Mahdavi, S.A.; Javadian, B.; Rezaei, R.; Moosazadeh, M.; Khademlou, M.; Seyedpour, S.H.; Syadatpanah, A. High Seroprevalence of *Toxoplasma gondii* Antibody in HIV/AIDS Individuals from North of Iran. *Iran J. Parasitol.* **2015**, *10*, 584–589.
67. Zaini, R.G.; Ismail, K.A.; Dahlawi, H. Seroprevalence of *Toxoplasma gondii* among AIDS Patients in Saudi Arabia. *World J. AIDS* **2016**, *6*, 81–86.

68. Rezanezhad, H.; Sayadi, F.; Shadmand, E.; Nasab, S.D.M.; Yazdi, H.R.; Solhjoo, K.; Kazemi, A.; Maleki, M.; Vasmehjani, A.A. Seroprevalence of *Toxoplasma gondii* among HIV Patients in Jahrom, Southern Iran. *Korean J. Parasitol.* **2017**, *55*, 99–103.
69. Aghaee, R.; Saki, S.S.; Didehdar, M.; Hajihossein, R.; Eslamirad, Z. Epidemiologic evaluation of toxoplasmosis and leading risk factors in HIV/AIDS patients in Arak City, Iran. *Australas Med. J.* **2017**, *10*, 865–869.
70. Aghakhani, A.; Velayati, A.A.; Bavand, A.; Banifazl, M.; Ramezani, A. Seroprevalence of *Toxoplasma gondii* in Iranian HIV Infected Patients. *ECCMID* **2018**, *28*, 1386.
71. Shafieenia, S.; Saki, J.; Khademvatan, S.; Moradi-Choghakabodi, P. Molecular and Serological Evaluation of Toxoplasmosis in AIDS Cases in Southwest Iran. *Jundishapur J. Microbiol.* **2018**, *11*.
72. Ebrahim-Saraie, H.S.; Heidari, H.; Mousavi, S.; Asefi, H.; Abadi, A.; Afsar-Kazerooni, P.; Motamedifar, M. *Toxoplasma gondii* seroprevalence and related risk factors in patients with HIV in Iran. *Arch. Hell. Med.* **2018**, *35*, 400–404.
73. Arefkhah, N.; Sarkari, B.; Afrashteh, M.; Rezaei, Z.; Dehghani, M. *Toxoplasma gondii*: The Prevalence and Risk Factors in HIV-Infected Patients in Fars Province, Southern Iran. *Iran Red. Crescent Med. J.* **2018**, *20*, e66521.
74. Nazari, N.; Bozorgomid, A.; Janbakhsh, A.; Bashiri, F. *Toxoplasma gondii* and human immunodeficiency virus co-infection in western Iran: A cross sectional study. *Asian Pac. J. Trop. Med.* **2018**, *11*, 58.
75. Bavand, A.; Aghakhani, A.; Mohraz, M.; Banifazl, M.; Karami, A.; Golkar, M.; Babaie, J.; Saleh, P.; Mamishi, S.; Ramezani, A. Prevalence of *Toxoplasma gondii* Antibodies and DNA in Iranian HIV Patients. *Iran J. Pathol.* **2019**, *14*, 68–75.
76. Ahmadpour, E.; Pishkarie-Asl, R.; Spotin, A.; Samadi, H.K.; Didarlu, H.; Azadi, Y.; Barac, A. Sero-molecular evaluation of *Toxoplasma gondii* infection among HIV-positive patients. *Trans. R. Soc. Trop. Med. Hyg.* **2019**, 113, 771–775.
77. Bokharaei-Salim, F.; Esteghamati, A.; Khanaliha, K.; Kalantari, S.; Sayyahfar, S.; Donyavi, T.; Garshasbi, S.; Asgari, Q.; Salemi, B. Evaluation of a PCR assay for diagnosis of toxoplasmosis in serum and peripheral blood mononuclear cell among HIV/AIDS patients. *J. Parasit. Dis.* **2020**, *44*, 159–165.
78. Hosseini, S.A.; Sharif, M.; Sarvi, S.; Abediankenari, S.; Hashemi-Soteh, M.B.; Amouei, A.; Montazeri, M.; Aghayan, S.A.; Gholami, S.; Shaker, D. Genetic characterization of *Toxoplasma gondii* in Iranian HIV positive patients using multilocus nested-PCR-RFLP method. *Parasitology* **2020**, *147*, 322–328.
79. Holliman, R.E. Serological study of the prevalence of toxoplasmosis in asymptomatic patients infected with human immunodeficiency virus. *Epidemiol. Infect.* **1990**, *105*, 415–418.
80. Aspock, H.; Hassl, A. Parasitic infections in HIV patients in Austria: first results of a long-term study. *Zentralbl Bakteriol.* **1990**, *272*, 540–546.
81. Zangerle, R.; Allerberger, F.; Pohl, P.; Fritsch, P.; Dierich, M.P. High risk of developing toxoplasmic encephalitis in AIDS patients seropositive to *Toxoplasma gondii*. *Med. Microbiol. Immunol.* **1991**, *180*, 59–66.
82. Sykora, J.; Zastera, M.; Stankova, M. Toxoplasmic antibodies in sera of HIV-infected persons. *Folia Parasitol.* **1992**, *39*, 177–180.
83. Zufferey, J.; Sugar, A.; Rudaz, P.; Bille, J.; Glauser, M.P.; Chave, J.P. Prevalence of latent toxoplasmosis and serological diagnosis of active infection in HIV-positive patients. *Eur. J. Clin. Microbiol. Infect. Dis.* **1993**, *12*, 591–595.
84. Huengsberg, M.; Jayaweera, D.T.; Wagstaffe, S.; Opaneye, A.A.; Wade, A.A.; Shahmanesh, M. *Toxoplasma* seroprevalence in HIV-positive patients in West Midlands. *Int. J. STD AIDS* **1995**, *6*, 223.
85. Champs, C.D.; Pelloux, H.; Cambon, M.; Fricker-Hidalgo, H.; Goullier-Fleuret, A.; Ambroise-Thomas, P. Evaluation of the second generation IMx Toxo IgG antibody assay for detection of antibodies to *Toxoplasma gondii* in human sera. *J. Clin. Lab. Anal.* **1997**, *11*, 214–219.
86. Djurković-Djaković, O.; Bobić, B.; Vuković, D.; Marinković, J.; Jevtović, D. Risk for toxoplasmic encephalitis in AIDS patients in Yugoslavia. *Int. J. Infect. Dis.* **1997**, *2*, 74–78.
87. Garly, M.L.; Petersen, E.; Pedersen, C.; Lundgren, J.D.; Gerstoft, J. Toxoplasmosis in Danish AIDS patients. *Scand. J. Infect. Dis.* **1997**, *29*, 597–600.
88. Reiter-Owona, I.; Bialek, R.; Rockstroh, J.; Seitz, H. The probability of acquiring primary *Toxoplasma* infection in HIV-infected patients: results of an 8-year retrospective study. *Infection* **1998**, *26*, 20–25.
89. Boto de los Bueis, A.; Vega Aleman, D.; Galvez Acebal, J.; Merino Munoz, D.; Creagh Cerquera, R.; Pujol de la Llave, E. Prevalence of latent *Toxoplasma* infection in HIV infection patients. *An. Med. Interna.* **1998**, *15*, 298–300.
90. Belanger, F.; Derouin, F.; Grangeot-Keros, L.; Meyer, L.; HEMOCO; Groups, S.S. Incidence and risk factors of toxoplasmosis in a cohort of human immunodeficiency virus-infected patients: 1988–1995. *Clin. Infect. Dis.* **1999**, *28*, 575–581.
91. San-Andrés, F.-J.; Rubio, R.; Castilla, J.; Pulido, F.; Palao, G.; de Pedro, I.; Costa, J.-R.; del Palacio, A. Incidence of acquired immunodeficiency syndrome-associated opportunistic diseases and the effect of treatment on a cohort of 1115 patients infected with human immunodeficiency virus, 1989–1997. *Clin. Infect. Dis.* **2003**, *36*, 1177–1185.
92. Llenas-García, J.; Rubio, R.; Hernando, A.; Fiorante, S.; Maseda, D.; Matarranz, M.; Costa, J.; Alonso, B.; Pulido, F. Clinico-epidemiological characteristics of HIV-positive immigrants: study of 371 cases. *Enferm Infecc. Microbiol. Clin.* **2012**, *30*, 441–451.
93. Csep, A.; Drăghici, S. *Toxoplasma gondii* infection in immunocompromised individuals. *BMC Infect. Dis.* **2013**, *13*, P4.
94. Kodym, P.; Malý, M.; Beran, O.; Jilich, D.; Rozsypal, H.; Machala, L.; Holub, M. Incidence, immunological and clinical characteristics of reactivation of latent *Toxoplasma gondii* infection in HIV-infected patients. *Epidemiol. Infect.* **2015**, *143*, 600–607.
95. Đaković-Rode, O.; Židovec-Lepej, S.; Vodnica Martucci, M.; Lasica Polanda, V.; Begovac, J. Prevalence of antibodies against *Toxoplasma gondii* in patients infected with human immunodeficiency virus in Croatia. *Infektološki Glasnik* **2010**, *30*, 5–10.
96. Aydın, Ö.A.; Karaosmanoğlu, H.K.; Korkusuz, R.; Nazlıcan, Ö. HIV/AIDS Hastalarında *Toxoplasma gondii* IgG Seroprevalansı. *Turkiye Parazitol. Derg.* **2011**, *35*, 65–67.
97. Ene, L.; Marcotte, T.D.; Umlauf, A.; Grancea, C.; Temereanca, A.; Bharti, A.; Achim, C.L.; Letendre, S.; Ruta, S.M. Latent toxoplasmosis is associated with neurocognitive impairment in young adults with and without chronic HIV infection. *J. Neuroimmunol.* **2016**, *299*, 1–7.
98. Senoglu, S.; Yesilbag, Z.; Altuntas Aydin, O.; Kumbasar Karaosmanoglu, H.; Kart Yasar, K. *Toxoplasma gondii* IgG Seroprevalence in Patients with HIV/AIDS. *Turkiye Parazitol. Derg.* **2018**, *42*, 175–179.
99. Israelski, D.M.; Chmiel, J.S.; Poggensee, L.; Phair, J.P.; Remington, J.S. Prevalence of *Toxoplasma* infection in a cohort of homosexual men at risk of AIDS and toxoplasmic encephalitis. *J. Acquir. Immune Defic .Syndr.* **1993**, *6*, 414–418.
100. Grant, I.H.; Gold, J.W.; Rosenblum, M.; Niedzwiecki, D.; Armstrong, D. *Toxoplasma gondii* serology in HIV-infected patients: the development of central nervous system toxoplasmosis in AIDS. *AIDS* **1990**, *4*, 519–521.
101. Wallace, M.R.; Rossetti, R.J.; Olson, P.E. Cats and toxoplasmosis risk in HIV-infected adults. *JAMA* **1993**, *269*, 76–77.
102. Fachado, A.; Fonte, L.; Alberti, E.; Hadad, P.; Fonseca, L.; Machin, R.; Finlay, C. Usefulness of the detection of *Toxoplasma gondii* antigens in AIDS patients. *Rev. Inst. Med. Trop. Sao Paulo* **1994**, *36*, 525–529.
103. Mathews, W.C.; Fullerton, S.C. Use of a clinical laboratory database to estimate *Toxoplasma* seroprevalence among human immunodeficiency virus-infected patients. Overcoming bias in secondary analysis of clinical records. *Arch. Pathol. Lab. Med.* **1994**, *118*, 807–810.
104. Galvan Ramirez, M.L.; Valdez Alvarado, V.; Vargas Gutierrez, G.; Jimenez Gonzalez, O.; Garcia Cosio, C.; Vielma Sandoval, M. Prevalence of IgG and IgM anti-*Toxoplasma* antibodies in patients with HIV and acquired immunodeficiency syndrome (AIDS). *Rev. Soc. Bras. Med. Trop.* **1997**, *30*, 465–467.
105. Gongora-Biachi, R.A.; Gonzalez-Martinez, P.; Castro-Sansores, C.; Alvarez-Moguel, R.; Pavia-Ruz, N.; Lara-Perera, D.; Alonzo-Salomon, G.; Palacios-Perez, E. [Antibodies against *Toxoplasma gondii* in patients with HIV in Yucatan]. *Rev. Invest. Clin.* **1998**, *50*, 419–422.

106. Johns, D.G.; Gill, M.J. Seroprevalence of cytomegalovirus, *Toxoplasma gondii*, syphilis, and hepatitis B and C virus infections in a regional population seropositive for HIV infection. *Can. J. Infect. Dis.* **1998**, *9*, 209–214.
107. Falusi, O.; French, A.L.; Seaberg, E.C.; Tien, P.C.; Watts, D.H.; Minkoff, H.; Piessens, E.; Kovacs, A.; Anastos, K.; Cohen, M.H. Prevalence and predictors of *Toxoplasma* seropositivity in women with and at risk for human immunodeficiency virus infection. *Clin. Infect. Dis.* **2002**, *35*, 1414–1417.
108. Bharti, A.R.; McCutchan, A.; Deutsch, R.; Smith, D.M.; Ellis, R.J.; Cherner, M.; Woods, S.P.; Heaton, R.K.; Grant, I.; Letendre, S.L. Latent *Toxoplasma* Infection and Higher *Toxoplasma gondii* Immunoglobulin G Levels Are Associated With Worse Neurocognitive Functioning in HIV-Infected Adults. *Clin. Infect. Dis.* **2016**, *63*, 1655–1660.
109. O'Bryan, T.A.; Okulicz, J.F.; Bradley, W.P.; Ganesan, A.; Merritt, S.E.; Agan, B.K. *Toxoplasma gondii* seroprevalence: 30-year trend in an HIV-infected US military cohort. *Diagn. Microbiol. Infect. Dis.* **2016**, *84*, 34–35.
110. Meisheri, Y.V.; Mehta, S.; Patel, U. A prospective study of seroprevalence of Toxoplasmosis in general population, and in HIV/AIDS patients in Bombay, India. *J. Postgrad. Med.* **1997**, *43*, 93–97.
111. Sukthana, Y.; Chintana, T.; Lekkla, A. *Toxoplasma gondii* antibody in HIV-infected persons. *J. Med. Assoc. Thai.* **2000**, *83*, 681–684.
112. Wanachiwanawin, D.; Sutthent, R.; Chokephalbulkit, K.; Mahakittikun, V.; Ongrotchanakun, J.; Monkong, N. *Toxoplasma gondii* antibodies in HIV and non-HIV infected Thai pregnant women. *Asian Pac. J. Allergy Immunol.* **2001**, *19*, 291.
113. Praharaj, C.A.; Singh, S.P.; Chander, C.Y.; Nagendra, A. Serological diagnosis of *Toxoplasma gondii* infection in various patient population in the armed forces. *Med. J. Armed Forces India* **2001**, *57*, 298–301.
114. Malla, N.; Sengupta, C.; Dubey, M.; Sud, A.; Dutta, U. Antigenaemia and antibody response to *Toxoplasma gondii* in human immunodeficiency virus-infected patients. *Br. J. Biomed. Sci.* **2005**, *62*, 19–23.
115. Sucilathangam, G.; Palaniappan, N.; Sreekumar, C.; Anna, T. Seroprevalence of *Toxoplasma gondii* in southern districts of Tamil Nadu using IgG-ELISA. *J. Parasit. Dis.* **2012**, *36*, 159–164.
116. Bhattacharyya, S.; Khurana, S.; Dubey, M.L. Anti-*Toxoplasma gondii* antibody detection in serum and urine samples by enzyme-linked immunosorbent assay in HIV-infected patients. *Indian J. Pathol. Microbiol.* **2013**, *56*, 20–23.
117. Anuradha, B.; Preethi, C. Seroprevalence of *Toxoplasma* IgG Antibodies in HIV Positive Patients in and Around Khammam, Telangana State. *J. Clin. Diagn. Res.* **2014**, *8*, l01–102.
118. Chemoh, W.; Sawangjaroen, N.; Siripaitoon, P.; Andiappan, H.; Hortiwakul, T.; Sermwittayawong, N.; Charoenmak, B.; Nissapatorn, V. *Toxoplasma gondii* - Prevalence and Risk Factors in HIV-infected Patients from Songklanagarind Hospital, Southern Thailand. *Front. Microbiol.* **2015**, *6*, 1304.
119. Haryati, S.; Prasetyo, A.A.; Sariyatun, R.; Sari, Y.; Murkati. Interferon-γ +874A/T polymorphism associated with *Toxoplasma gondii* seropositivity in HIV patients. *Asian Pac. J. Trop. Dis.* **2015**, *5*, 798–803.
120. Prasetyo, A.A.; Sariyatun, R.; Reviono; Sari, Y.; Hudiyono; Haryati, S.; Adnan, Z.A.; Hartono; Kageyama, S. The APOBEC3B deletion polymorphism is associated with prevalence of hepatitis B virus, hepatitis C virus, Torque Teno virus, and *Toxoplasma gondii* co-infection among HIV-infected individuals. *J. Clin. Virol.* **2015**, *70*, 67–71.
121. Sari, Y.; Haryati, S.; Raharjo, I.; Prasetyo, A.A. *Toxoplasma* and viral antibodies among HIV patients and inmates in central Java, Indonesia. *Southeast Asian J. Trop. Med. Public Health* **2015**, *46*, 977–985.
122. Uppal, B.; Aggarwal, P.; Perween, N.; Sud, A. Seroprevalence of *Toxoplasma* among HIV infected and HIV non-infected individuals in North India. *Asian Pac. J. Trop. Dis.* **2015**, *5*, S15–S18.
123. Singh, L.; Mishra, S.; Prasanna, S.; Cariappa, M.P. Seroprevalence of TORCH infections in antenatal and HIV positive patient populations. *Med. J. Armed Forces India* **2015**, *71*, 135–138.
124. Kasthuri, A. A prospective cohort study on incidence of "opportunistic infections" among HIV infected ART naïve rural people of Kanchipuram district. *Int. J. Community Med. Public Health* **2018**, *5*, 2770–2774.
125. Pimpalkar, S.; Mishra, M.; Kurhade, A.; Unakal, C.; Kurhade, G.; Powar, R. Prevalence of Toxoplasmosis among HIV/AIDS Patients and Correlation of Radiological Investigations with Laboratory Findings. *Microbiol. Res. J. Int.* **2018**, *26*, 1–10.
126. Garg, S.; Sharma, B.; Bithu, R.; Vyas, N. Seroprevalence of IgM and IgG antibodies to *Toxoplasma* infection in human immunodeficiency virus-positive antiretroviral therapy-naive individuals. *Muller J. Med. Sci. Res.* **2019**, *10*, 8–12.
127. Halleyantoro, R.; Andriyani, Y.; Sari, I.P.; Kurniawan, A. Nested PCR methods for detection *Toxoplasma gondii* B1 gene in Cerebrospinal Fluid of HIV patients. *J. Biomed. Transl. Res.* **2019**, *5*, 62–66.
128. Yoong, K.-Y.; Cheong, I. A study of Malaysian drug addicts with human immunodeficiency virus infection. *Int. J. STD AIDS* **1997**, *8*, 118-123.
129. Oh, M.-d.; Park, S.W.; Kim, H.B.; Kim, U.S.; Kim, N.J.; Choi, H.J.; Shin, D.H.; Lee, J.S.; Choe, K. Spectrum of opportunistic infections and malignancies in patients with human immunodeficiency virus infection in South Korea. *Clin. Infect. Dis.* **1999**, *29*, 1524–1528.
130. Hagiwara, E.; Ito, A.; Shirai, A.; Kawada, K.; Okubo, T.; Amano, T.; Ishigatsubo, Y. Seroprevalence of anti-*Toxoplasma* IgG antibody in Japanese patients with HIV infection. *Kansenshogaku Zasshi* **2001**, *75*, 703.
131. Zhou, M.; Huang, X.; Ku, D. Survey of co-infection with HIV and *Toxoplasma gondii* among Xinjiang People. *Chin. J. Zoonosis.* **2001**, *17*, 127.
132. Nissapatorn, V.; Kamarulzaman, A.; Init, I.; Tan, L.H.; Rohela, M.; Norliza, A.; Chan, L.L.; Latt, H.M.; Anuar, A.K.; Quek, K.F. Seroepidemiology of toxoplasmosis among HIV-infected patients and healthy blood donors. *Med. J. Malaysia* **2002**, *57*, 304–310.
133. Nissapatorn, V.; Lee, C.K.; Cho, S.M.; Rohela, M.; Anuar, A.K.; Quek, K.F.; Latt, H.M. Toxoplasmosis in HIV/AIDS patients in Malaysia. *Southeast Asian J. Trop. Med. Public Health* **2003**, *34 Suppl 2*, 80–85.
134. Nissapatorn, V.; Lee, C.; Quek, K.F.; Leong, C.L.; Mahmud, R.; Abdullah, K.A. Toxoplasmosis in HIV/AIDS patients: a current situation. *Jpn. J. Infect. Dis.* **2004**, *57*, 160–165.
135. Hung, C.-C.; Chen, M.; Hsieh, S.; Hsiao, C.; Sheng, W.-H.; Chang, S.-C. Prevalence of *Toxoplasma gondii* infection and incidence of *Toxoplasma* encephalitis in non-haemophiliac HIV-1-infected adults in Taiwan. *Int. J. STD AIDS* **2005**, *16*, 302–306.
136. Naito, T.; Inui, A.; Kudo, N.; Matsumoto, N.; Fukuda, H.; Isonuma, H.; Sekigawa, I.; Dambara, T.; Hayashida, Y. Seroprevalence of IgG anti-*Toxoplasma* antibodies in asymptomatic patients infected with human immunodeficiency virus in Japan. *Intern. Med.* **2007**, *46*, 1149–1150.
137. Zhang, X.; Xu, J.; Peng, H.; Ma, Y.; Han, L.; Ruan, Y.; Su, B.; Wang, N.; Shao, Y. HCV coinfection associated with slower disease progression in HIV-infected former plasma donors naive to ART. *PLoS One.* **2008**, *3*.
138. Hua, H.; Huan, X.; Dong, M.; al., e. Survey on infection of *Toxoplasma gondii* in HIV/AIDS patients. *Chin. J. Mod. Med.* **2009**, *11*, 47–49.
139. Tian, L.; Cheng, G.; Chen, J.; al., e. Survey on coinfection with *Toxoplasma* and HIV among rural people in China. *Chin. J. Schisto. Control* **2010**, *22*.
140. Song, R. Relationship between *Toxoplasma* IgG and routine screening items of infectious diseases of blood donors. *Chin. J. Schisto. Control* **2012**, *24*, 371–372.
141. You, Y.; Li, W.; Shen, L.; Nie, D. Serological investigation of *Toxoplasma gondii* infection in HIV positive cases in Dali and Dehong of Yunnan. *Chinese J. Parasitol. Parasit. Dis.* **2012**, *30*, 418–419.
142. John, L.N.; McBride, W.J.; Millan, J.; Wilson, K. Seroprevalence of anti-*Toxoplasma gondii* antibodies in HIV/AIDS patients and healthy blood donors at the Port Moresby General Hospital, Papua New Guinea. *PNG Med. J.* **2012**, *55*, 88–93.
143. Lim, R.B.; Tan, M.T.; Young, B.; Lee, C.C.; Leo, Y.S.; Chua, A.; Ng, O.T. Risk factors and time-trends of cytomegalovirus (CMV), syphilis, toxoplasmosis and viral hepatitis infection and seroprevalence in human immunodeficiency virus (HIV) infected patients. *Ann. Acad. Med. Singapore* **2013**, *42*, 667–673.

144. Takahashi, H.; Tsubouchi, A.; Murai, K.; Yamanouchi, M.; Tanei, M.; Sakamoto, R.; Uehara, Y.; Fan, C.; Naito, T. Seroprevalence of *Toxoplasma gondii* infection among HIV-infected patients in Tokyo, Japan. *Clin. Res. HIV/AIDS* **2014**, *1*, 1007.
145. Pang, X.; Chen, S.; Gao, K.; al, e. Serum epidemiological analysis of opportunistic infection of pathogenic protozoa in HIV/AIDS. *J. Trop. Med.* **2015**, *15*, 1425.
146. Angal, L.; Lim, Y.; Yap, N.; Ngui, R.; Amir, A.; Kamarulzaman, A.; Rohela, M. Toxoplasmosis in HIV and non HIV prisoners in Malaysia. *Trop. Biomed.* **2016**, *33*, 159–169.
147. Chen, X.; Ye, M.; Zhou, Y.H.; Liu, F.L.; Duo, L.; Li, H.; Pang, W.; Zheng, Y.T. High seroprevalence of *Toxoplasma gondii* and HIV-1 co-infection among drug users in Yunnan province, southwest China. *Sci. China Life Sci.* **2016**, *59*, 857–859.
148. Shen, G.; Wang, X.; Sun, H.; Gao, Y. Seroprevalence of *Toxoplasma gondii* infection among HIV/AIDS patients in Eastern China. *Korean J. Parasitol.* **2016**, *54*, 93–96.
149. Pang, W.; Shang, P.; Li, Q.; Xu, J.; Bi, L.; Zhong, J.; Pei, X. Prevalence of Opportunistic Infections and Causes of Death among Hospitalized HIV-Infected Patients in Sichuan, China. *Tohoku J. Exp. Med.* **2018**, *244*, 231–242.
150. Hoshina, T.; Horino, T.; Saiki, E.; Aonuma, H.; Sawaki, K.; Miyajima, M.; Lee, K.; Nakaharai, K.; Shimizu, A.; Hosaka, Y. Seroprevalence and associated factors of *Toxoplasma gondii* among HIV-infected patients in Tokyo: A cross sectional study. *J. Infect. Chemother.* **2020**, *26*, 33–37.

Section/topic	#	Checklist item	Reported on page #
**TITLE**			
Title	1	Identify the report as a systematic review, meta-analysis, or both.	Title, page 1
**ABSTRACT**			
Structured summary	2	Provide a structured summary including, as applicable: background; objectives; data sources; study eligibility criteria, participants, and interventions; study appraisal and synthesis methods; results; limitations; conclusions and implications of key findings; systematic review registration number.	Abstract, pages 2 and 3
**INTRODUCTION**			
Rationale	3	Describe the rationale for the review in the context of what is already known.	Introduction, paragraphs 1 and 2
Objectives	4	Provide an explicit statement of questions being addressed with reference to participants, interventions, comparisons, outcomes, and study design (PICOS).	Introduction
**METHODS**			
Protocol and registration	5	Indicate if a review protocol exists, if and where it can be accessed (e.g., Web address), and, if available, provide registration information including registration number.	there is no published protocol
Eligibility criteria	6	Specify study characteristics (e.g., PICOS, length of follow-up) and report characteristics (e.g., years considered, language, publication status) used as criteria for eligibility, giving rationale.	Method, section 2.1
Information sources	7	Describe all information sources (e.g., databases with dates of coverage, contact with study authors to identify additional studies) in the search and date last searched.	Method, section 2.1
Search	8	Present full electronic search strategy for at least one database, including any limits used, such that it could be repeated.	Supplementary Figure 1
Study selection	9	State the process for selecting studies (i.e., screening, eligibility, included in systematic review, and, if applicable, included in the meta-analysis).	Method, section 2.1
Data collection process	10	Describe method of data extraction from reports (e.g., piloted forms, independently, in duplicate) and any processes for obtaining and confirming data from investigators.	Method, section 2.2
Data items	11	List and define all variables for which data were sought (e.g., PICOS, funding sources) and any assumptions and simplifications made.	Method, section 2.2

Risk of bias in individual studies	12	Describe methods used for assessing risk of bias of individual studies (including specification of whether this was done at the study or outcome level), and how this information is to be used in any data synthesis.	Method, section 2.2
Summary measures	13	State the principal summary measures (e.g., risk ratio, difference in means).	Method, section 2.3
Synthesis of results	14	Describe the methods of handling data and combining results of studies, if done, including measures of consistency (e.g., I^2) for each meta-analysis.	Method, section 2.3

Section/topic	#	Checklist item	Reported on page #
Risk of bias across studies	15	Specify any assessment of risk of bias that may affect the cumulative evidence (e.g., publication bias, selective reporting within studies).	Method, section 2.3
Additional analyses	16	Describe methods of additional analyses (e.g., sensitivity or subgroup analyses, meta-regression), if done, indicating which were pre-specified.	Method, section 2.3
**RESULTS**			
Study selection	17	Give numbers of studies screened, assessed for eligibility, and included in the review, with reasons for exclusions at each stage, ideally with a flow diagram.	Results, section 3.1 Supplementary figure 2
Study characteristics	18	For each study, present characteristics for which data were extracted (e.g., study size, PICOS, follow-up period) and provide the citations.	Results, section 3.1 supplementary table 1
Risk of bias within studies	19	Present data on risk of bias of each study and, if available, any outcome level assessment (see item 12).	Results, table2
Results of individual studies	20	For all outcomes considered (benefits or harms), present, for each study: (a) simple summary data for each intervention group (b) effect estimates and confidence intervals, ideally with a forest plot.	Results, Table 1, and 2
Synthesis of results	21	Present results of each meta-analysis done, including confidence intervals and measures of consistency.	Results, tables
Risk of bias across studies	22	Present results of any assessment of risk of bias across studies (see Item 15).	Not applicable
Additional analysis	23	Give results of additional analyses, if done (e.g., sensitivity or subgroup analyses, meta-regression [see Item 16]).	results and table 2
**DISCUSSION**			
Summary of evidence	24	Summarize the main findings including the strength of evidence for each main outcome; consider their relevance to key groups (e.g., healthcare providers, users, and policy makers).	Discussion, Paragraphs 1
Limitations	25	Discuss limitations at study and outcome level (e.g., risk of bias), and at review-level (e.g., incomplete retrieval of identified research, reporting bias).	Discussion, Paragraphs 4
Conclusions	26	Provide a general interpretation of the results in the context of other evidence, and implications for future research.	Discussion, Paragraphs 5
**FUNDING**			
Funding	27	Describe sources of funding for the systematic review and other support (e.g., supply of data); role of funders for the systematic review.	Method, paragraph 1

PRISMA Checklist From: Moher D, Liberati A, Tetzlaff J, Altman DG, The PRISMA Group (2009). Preferred Reporting Items for Systematic Reviews and Meta-Analyses: The PRISMA Statement. PLoS Med 6(7): e1000097. doi:10.1371/journal.pmed1000097

For more information, visit: **www.prisma-statement.org**.
